# Degradome sequencing reveals an integrative miRNA-mediated gene interaction network regulating rice seed vigor

**DOI:** 10.1186/s12870-022-03645-2

**Published:** 2022-06-01

**Authors:** Shiqi Zhou, Kerui Huang, Yan Zhou, Yingqian Hu, Yuchao Xiao, Ting Chen, Mengqi Yin, Yan Liu, Mengliang Xu, Xiaocheng Jiang

**Affiliations:** 1 College of Life Sciences, Hunan Normal University, Changsha, 410081 China; 2 Hunan Province Key Laboratory of Crop Sterile Germplasm Resource Innovation and Application, Changsha, 410081 China; 3 College of Life and Environmental Sciences, Hunan University of Arts and Science, Changde, 415000 China

**Keywords:** *Oryza sativa* L., Seed vigor, miRNAs, Degradome, Gene interaction network

## Abstract

**Background:**

It is well known that seed vigor is essential for agricultural production and rice (*Oryza sativa* L.) is one of the most important crops in the world. Though we previously reported that miR164c regulates rice seed vigor, but whether and how other miRNAs cooperate with miR164c to regulate seed vigor is still unknown.

**Results:**

Based on degradome data of six RNA samples isolated from seeds of the wild-type (WT) *indica* rice cultivar ‘Kasalath’ as well as two modified lines in ‘Kasalath’ background (miR164c-silenced line [*MIM164c*] and miR164c overexpression line [*OE164c*]), which were subjected to either no aging treatment or an 8-day artificial aging treatment, 1247 different target transcripts potentially cleaved by 421 miRNAs were identified. The miRNA target genes were functionally annotated via GO and KEGG enrichment analyses. By STRING database assay, a miRNA-mediated gene interaction network regulating seed vigor in rice was revealed, which comprised at least four interconnected pathways: the miR5075-mediated oxidoreductase related pathway, the plant hormone related pathway, the miR164e related pathway, and the previously reported *RPS27AA* related pathway. Knockout and overexpression of the target gene *Os02g0817500* of miR5075 decreased and enhanced seed vigor, respectively. By Y2H assay, the proteins encoded by five seed vigor-related genes, *Os08g0295100*, *Os07g0633100*, *REFA1*, *OsPER1* and *OsGAPC3,* were identified to interact with Os02g0817500.

**Conclusions:**

miRNAs cooperate to regulate seed vigor in rice via an integrative gene interaction network comprising miRNA target genes and other functional genes. The result provided a basis for fully understanding the molecular mechanisms of seed vigor regulation.

**Supplementary Information:**

The online version contains supplementary material available at 10.1186/s12870-022-03645-2.

## Background

Seed vigor, which refers to the potential of seed to germinate rapidly and uniformly under a wide range of field conditions, is essential for agricultural production [1, 2]. Seeds with high vigor germinate early, emerge neatly and quickly, have strong resistance to adverse environments, have obvious growth advantages and high production potential. During postharvest storage, the seed coat gradually loses its luster, and the seed germination rate and speed decrease. This process is called seed aging or deterioration [3]. A series of physiological and biochemical changes occur during seed aging, such as increase in cell membrane permeability, accumulation of reactive oxygen species (ROS), damage to mitochondria, changes in the antioxidant system and lipid peroxidation, DNA methylation and changes in organellar and nuclear genomes [4]. Because seed aging affects plant growth and consequently agricultural production, extensive research is being conducted to understand the mechanism governing seed vigor regulation. With the development and application of relevant techniques such as quantitative trait locus (QTL) mapping, transcriptomics, and proteomics, a large number of genes and proteins involved in the regulation of seed vigor have been identified [5].

MicroRNAs (miRNAs) are a class of non-coding 20–24-nt small RNAs that regulate various physiological processes, including growth, development, and stress resistance, mainly by degrading target transcripts or repressing their translation [6]. Previous studies have shown that miR164c plays multiple roles in regulating plant physiological processes. In *Arabidopsis*, miR164c controls petal number in a nonredundant manner by regulating the accumulation of *CUC1* and *CUC2* transcripts [7], and is considered as one of the candidate miRNAs involved in the response to iron deficiency [8]. Rice (*Oryza sativa* L.) is one of the most important crops in the world [9]. Inhibition of miR164c expression can improve the vigor and anti-aging ability of rice seeds [10]. Additionally, miR164c affects the key gene *RPS27AA* by acting on target genes *OsPSK5* and *TIL1* (*OMTN2*), which then affects energy metabolism-, endoplasmic reticulum (ER)-, stress-, and embryo development-related proteins, serine endopeptidase inhibitors and others, ultimately regulating rice seed vigor [11]. However, whether and how other miRNAs cooperate with miR164c to regulate seed vigor remains unknown.

A certain gene or gene family may be regulated by multiple miRNAs with different physiological effects. For example, the *MYB*2 gene promotes fiber development in cotton (*Gossypium hirsutum*), and is functionally homologous to *Arabidopsis thaliana GLABROUS1* (*GL1*), which is involved in trichome formation. Among the two *MYB2* homologs in cotton (AADD genome; *GhMYB2A* and *GhMYB2D*), *GhMYB2D* mRNA accumulates to a higher level than *GhMYB2A* mRNA during fiber initiation, and is targeted by miR828 and miR858. Overexpression of *GhMYB2A*, but not that of *GhMYB2D*, complements the *Arabidopsis gl1* mutant phenotype [12]. *MYB* is also a target gene of maize (*Zea mays* L.) miR159d (zma-miR159d), and is involved in maize leaf senescence regulation [13]. In *Arabidopsis*, abscisic acid (ABA)-induced accumulation of miR159 is a homeostatic mechanism to direct *MYB33* and *MYB101* transcript degradation and desensitize hormone signaling during seedling stress responses [14]. On the other hand, a given miRNA can also regulate multiple target genes to perform different functions. For example, multiple *Auxin Response Factor* (*ARF*) genes are documented targets of miR167. The enhanced miR167 level in transgenic rice overexpressing miR167 results in a substantial decline in the mRNA levels of four *OsARF* genes, which mediate the auxin response to contribute to normal plant growth and development, resulting in short-statured transgenic plants, with remarkably reduced tiller number [15]. *Arabidopsis* miR167 also regulates lateral root growth in response to nitrogen. Treatment of *Arabidopsis* seedlings with ammonium succinate reduces the miR167a/b level and increases *ARF8* expression in the pericycle and root cap, resulting in the initiation of lateral root formation [16]. In *Arabidopsis*, miR167 is also essential for the correct patterning of gene expression and the fertility of ovules and anthers. For example, *ARF6* and *ARF8* regulate gynoecium and stamen development in immature flowers. Pollen grows poorly in *arf6 arf8* gynoecia, and the miR167 overexpression line mimics the *arf6 arf8* phenotype. Consequently, ovule integuments cease to grow, while anthers grow abnormally but fail to release pollen [17]. Moreover, *Arabidopsis mARF6* and *mARF8* plants, with mutated miR167 target sites, exhibit defects in anther dehiscence and ovule development. The miR167a null mutant recapitulates *mARF6* or *mARF8* anther and ovule phenotypes. miR167-mediated anther growth arrest permits anther dehiscence; however, in the absence of miR167-mediated regulation, excess anther growth delays dehiscence by prolonging desiccation [18].

Degradome sequencing is a high-throughput technique based on parallel analysis of RNA ends (PARE), which has successfully been used to identify new miRNAs and their target genes [19], assess miRNA self-regulation [20], and characterize the relationship between miRNA and their target genes [21]. Degradome sequencing has enabled the identification of miRNAs and target genes related to plant growth and development, biotic and abiotic stress resistance and terpenoid biosynthesis in rice [22], *Arabidopsis* [19], *Populus* [23], and *Camellia sinensis* [24]. Gong et al. [25] reported miRNAs and their target genes involved in the regulation of seed vigor in sweet corn. In the present study, we used unaged and aged seeds of the wild-type (WT) *indica* rice cultivar ‘Kasalath’ and its miR164c-silenced (*MIM164c*) and overexpression (*OE164c*) lines for degradome sequencing to gain a general profile of the differences in miRNA and degrading target transcript (degradome transcript) levels among the different genotypes to reveal the miR164c-controlled gene interaction network that regulates seed vigor. The findings of this study provide new information on the molecular mechanisms involved in the regulation of seed vigor in rice.

## Results

### miR164c expression was negatively correlated with seed vigor

**Fig. 1:**
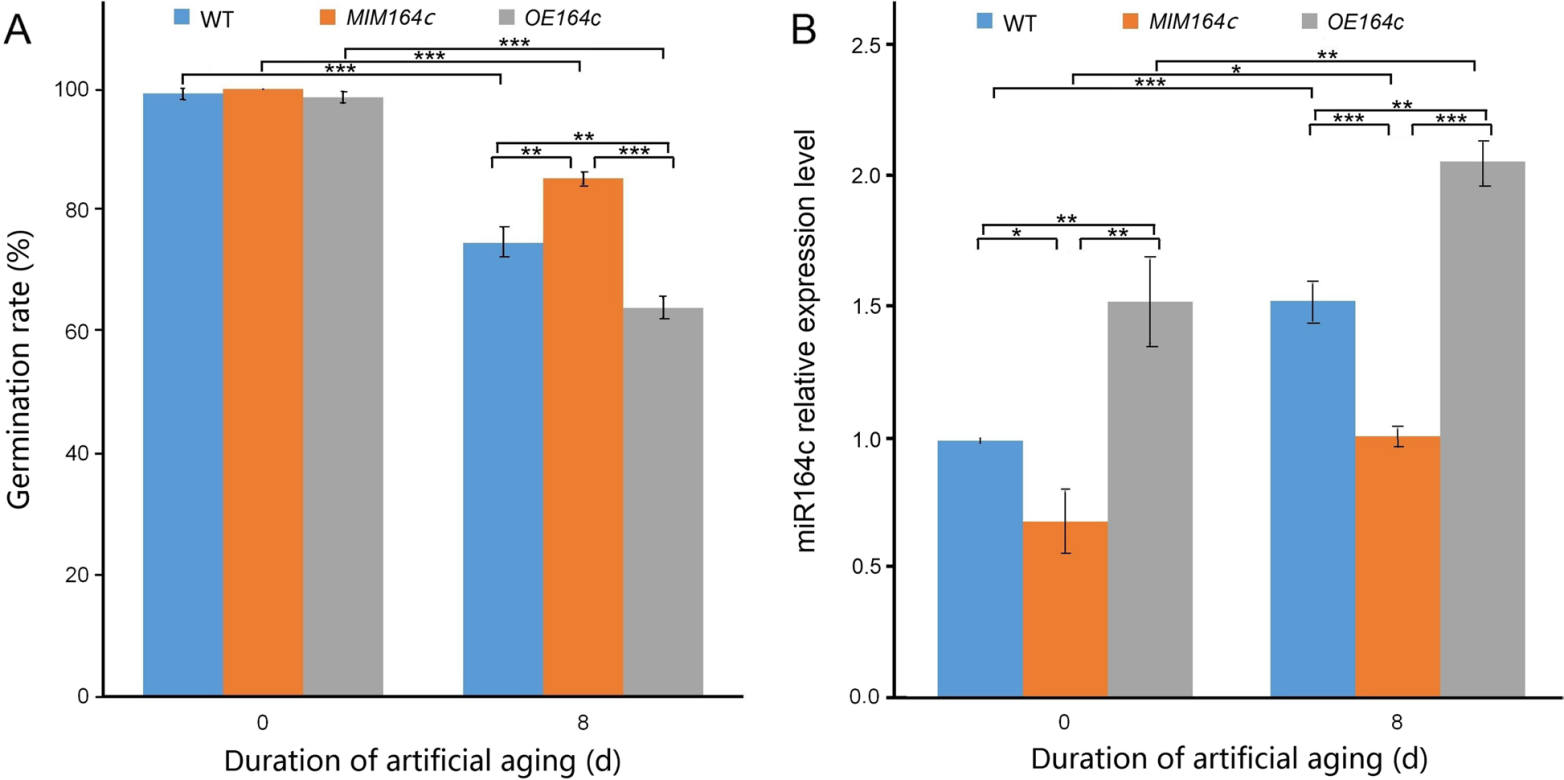
Characterization of unaged and artificially aged seeds of different genotypes of rice. **A** Seed germination rate; **B** RT-qPCR analysis of the expression level of miR164c. Data represent mean ± standard deviation (SD; *n* = 3). Significant differences among samples were determined using Student’s *t*-test (*P < 0.05, **P < 0.01, ***P < 0.001). In (**B**), The expression level of miR164c in unaged WT seeds was set as 1. WT indicates the wild-type rice cultivar ‘Kasalath’; *MIM164c* and *OE164c* indicate two modified lines in ‘Kasalath’ background, miR164c-silenced line ‘L13–1–2-1’ and miR164c overexpression line ‘L4–1–3-1’, respectively

Seed germination rate is an important indicator of seed vigor. The results of RT-qPCR analysis and germination test indicated that after 8 days of artificial aging, the expression level of miR164c and germination rates of WT, *MIM164c* and *OE164c* seeds differed significantly, consistent with previous studies [10, 11]. Regardless of aging, *OE164c* seeds showed the highest miR164c expression level and the lowest germination rate, whereas *MIM164c* seeds displayed the lowest miR164c expression level and the highest germination rate (Fig. 1).

### Differential expression profiles of miRNAs and target genes

Degradome sequencing assays of unaged WT, *MIM164c*, and *OE164c* seeds generated 23,290,765, 39,475,861, and 105,051,201 raw sequence reads, respectively, of which 5,910,027, 7,499,373, and 14,920,325 were unique, respectively. Similarly, degradome sequencing of artificially aged WT, *MIM164c*, and *OE164c* seeds generated 11,574,728, 23,358,523, and 75,229,379 raw sequence reads, of which 4,409,611, 6,992,505, and 14,893,430 were unique, respectively. Following BLAST analyses, 3,675,827 (62.20%), 4,501,572 (60.03%), and 7,887,621 (52.86%) unique reads in unaged WT, *MIM164c*, and *OE164c* seeds, respectively, and 2,797,531 (63.44%), 3,961,237 (56.65%), and 8,498,489 (57.06%) unique reads in aged WT, *MIM164c*, and *OE164c* seeds, respectively, could be matched with rice mRNAs (Table 1). The results indicated that both of *MIM164c* and *OE164c* seeds had higher raw as well as unique read counts than that of WT, which suggested that both miR164c-silence and miR164c-overexpression genetic transformation may lead to an increase in the degradome read counts in *MIM164c* and *OE164c* seeds. Especially, *OE164c* seeds had the highest read counts, which implied that the overexpression of miR164c exacerbated the cleavage of transcripts by miRNAs in seeds.

**Table 1:** Summary of the lllumina degradome sequencing data of unaged and artificially aged WT, *MIM164c*, and *OE164c* seeds

Variable	A^a^-WT		A-***MIM164c***		A-***OE164c***		B^b^-WT		B-***MIM164c***		B-***OE164c***		Total	
	Number	%	Number	%	Number	%	Number	%	Number	%	Number	%	Number	%
Raw reads	23,290,765	n.a.^c^	39,475,861	n.a.	105,051,201	n.a.	11,574,728	n.a.	23,358,523	n.a.	75,229,379	n.a.	277,980,457	n.a.
Reads< 15 nt in length after removing the 3′-adaptor sequences	115,963	0.5	150,392	0.38	438,675	0.42	52,118	0.45	114,625	0.49	291,424	0.39	1,163,197	0.42
Mappable reads	23,174,802	99.5	39,325,469	99.62	104,612,526	99.58	11,522,610	99.55	23,243,898	99.51	74,937,955	99.61	276,817,260	99.58
Transcript mapped reads	13,086,100	56.19	19,232,591	48.72	49,082,507	46.72	6,072,389	52.46	12,433,643	53.23	35,928,730	47.76	135,835,960	48.8
Unique raw reads	5,910,027	n.a.	7,499,373	n.a.	14,920,325	n.a.	4,409,611	n.a.	6,992,505	n.a.	14,893,430	n.a.	35,326,716	n.a.
Unique reads < 15 nt in length after removing three adaptor sequences	31,617	0.53	41,124	0.55	77,208	0.52	24,911	0.56	37,938	0.54	80,857	0.54	181,550	0.51
Unique mappable reads	5,878,410	99.47	7,458,249	99.45	14,843,117	99.48	4,384,700	99.44	69,54,567	99.46	14,812,573	99.46	35,145,166	99.49
Unique transcript mapped reads	3,675,827	62.2	4,501,572	60.03	7,887,621	52.86	2,797,531	63.44	3,961,237	56.65	8,498,489	57.06	17,031,820	48.21
No. of input transcripts	42,387	n.a.	42,387	n.a.	42,387	n.a.	42,387	n.a.	42,387	n.a.	42,387	n.a.	42,387	n.a.
No. of covered transcripts	33,806	79.76	33,227	78.39	36,332	85.71	34,687	81.83	35,095	82.8	38,044	89.75	39,538	93.28

^a^A indicates unaged seeds

^b^B indicates artificially aged seeds

^c^n.a. indicates not applicable

**Fig. 2:**
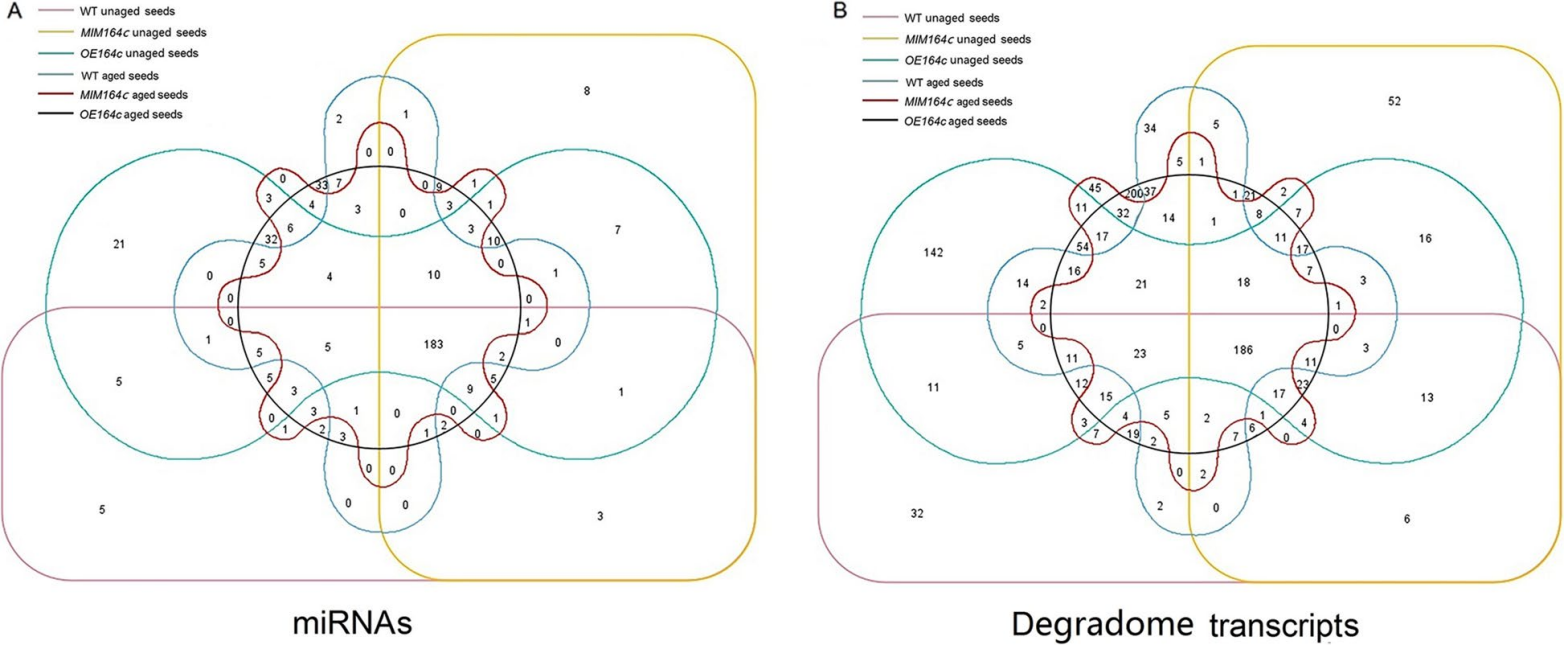
Venn diagram showing the numbers of miRNAs and degradome transcripts in the six seed samples. (**A**, **B**) Numbers of miRNAs (**A**) and degradome transcripts (**B**) overlapping among the six samples. A1, A2, and A3 indicate unaged WT, *MIM164c* and *OE164c* seeds, respectively; B1, B2, and B3 represent artificially aged WT, *MIM164c*, and *OE164c* seeds, respectively

A total of 1247 different degradome transcripts potentially cleaved by 421 miRNAs were identified, implying that a single miRNA targets more than one gene. A total of 186 degradome transcripts corresponding to 183 miRNAs were found in all six samples. However, the number of miRNAs and degradome transcripts differed among the WT, *MIM164c* and *OE164c* genotypes, regardless of aging. The number of miRNAs and degradome transcripts was the highest in unaged and aged *OE164c* seeds, up to 142 and 200 unique degradome transcripts were identified in unaged and artificially aged *OE164c* seeds, respectively (Fig. 2).

A total of eight target genes of miR164c were identified in all six samples, but the TPB value of miR164c target genes and the corresponding degradome transcripts differed among the different genotypes (Table 2). In unaged and aged WT and *OE164c* seeds as well as in aged *MIM164c* seeds, compared with other miRNAs, miR5075 had the highest number of degradome transcripts; in artificially aged *OE164c* seeds, the number of miR5075-related degradome transcripts was approximately 2-fold higher than that in other samples. In addition, a target transcript could be cleaved by 1–5 different miRNAs, such as the Os01t0180800–01 transcript was simultaneously targeted by miR414 and miR396a–c (Table S2).

**Table 2:** Degradome transcripts per billion (TPB) values of target genes of miR164c identified in unaged and artificially aged WT, *MIM164c*, and *OE164c* seeds

miR164c target gene	A^a^-WT	A-***MIM164c***	A-***OE164c***	B^b^-WT	B-***MIM164c***	B-***OE164c***
*Os02g0436400*	0	25.3	28.6	172.8	0	39.9
*OMTN1*	171.7	202.7	47.6	777.6	42.8	425.4
*OMTN2*	21.5	63.3	38.1	1295.9	321.1	551.6
*OMTN4*	300.5	25.3	85.7	691.2	984.7	478.5
*OMTN5*	42.9	25.3	0	259.2	85.6	332.3
*OMTN6*	0	0	9.5	129.6	42.8	19.9
*OMTN3*	0	0	38.1	432.0	42.8	239.3
*OsPSK5*	1803.3	658.6	980.5	345.6	1155.9	452.0

^a^A indicates unaged seeds

^b^B indicates artificially aged seeds

**Fig. 3:**
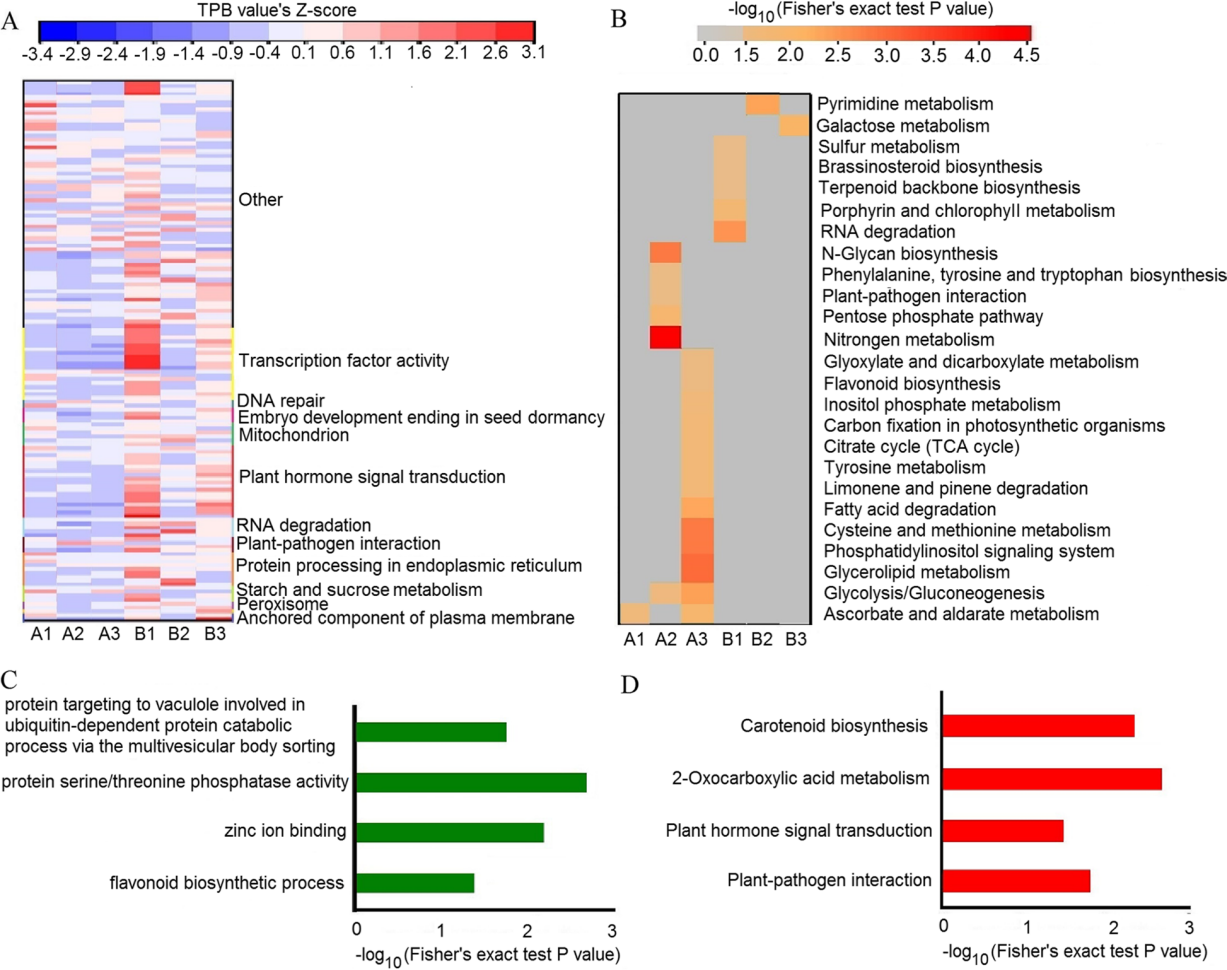
Functional cluster analysis and Gene Ontology (GO) and Kyoto Encyclopedia of Genes and Genomes (KEGG) enrichment analyses of target genes corresponding to the degradome transcripts. (**A**) Comparison of the abundance of degradome transcripts and TPB value based on the zero-mean normalization analysis of degradome transcripts in unaged and artificially aged WT, *MIM164c* and *OE164c* seeds. (**B**) KEGG enrichments of degradome transcripts unique to unaged and artificially aged WT, *MIM164c*, and *OE164c* seeds. (**C**) GO and (**D**) KEGG enrichments of target genes common to unaged and artificially aged WT and *OE164c* seeds but not to unaged and artificially aged *MIM164c* seeds. In (**A**) and (**B**), the greater the intensity of the red color, the higher the abundance of degradome transcripts; and the greater the intensity of the blue color, the lower the abundance of degradome transcripts. A1, A2, and A3 indicate unaged WT, *MIM164c* and *OE164c* seeds, respectively; B1, B2, and B3 represent artificially aged WT, *MIM164c*, and *OE164c* seeds, respectively

The TPB value of degradome transcripts of the miRNAs common to all six samples varied among the genotypes. It is worth noting that each genotype had the lower TPB value of degradome transcripts of miRNAs related to plant hormone signal transduction in unaged seeds than that in artificially aged seeds, and WT and *OE164c* artificially aged seeds had higher TPB value than that of *MIM164c* seeds (Fig. 3A, Table 3).

**Table 3:** Target genes that directly interacted with miR164c’ targets and involved in the plant hormone related pathway and degradome transcripts per billion (TPB) values identified in unaged and artificially aged WT, *MIM164c*, and *OE164c* seeds

miRNA	Target gene	A^a^-WT	A-***MIM164c***	A-***OE164c***	B^b^-WT	B-***MIM164c***	B-***OE164c***
miR172a-d	*RSR1*	0	0	95.2	31,275	12,201.1	33,577.3
miR160a-f	*OsARF8*	901.6	532	866.2	7775.6	642.2	6885.6
	*OsARF18*	14,726.9	9144.8	11,356.4	26,436.9	3382.1	13,611.7
	*OsARF22*	11,292	7092.9	10,014.2	28,078.4	6806.9	22,398.2
miR393a,b	*OsTIR1*	687	126.7	171.3	3110.2	1455.6	1528.7
miR172d	*Os06g0154200*	0	0	14.3	0	0	0
miR171a-i	*Os02g0663100*	42.9	50.7	133.3	864	128.4	478.5
miR167a-c	*OsARF12*	42.9	118.2	88.8	201.6	42.8	159.5
miR5075	*OsNAC52*	42.9	25.3	38.1	259.2	0	39.9
miR812a-e	*MOC1*	0	0	38.1	0	0	46.5
miR529a	*Os07g0107800*	0	0	0	0	0	13.3
miR156c,e	*MYB*	0	0	38.1	0	0	0

^a^A indicates unaged seeds

^b^B indicates artificially aged seeds

### GO and KEGG enrichment analyses of rice seed vigor-related miRNA target genes

Based on the annotated target transcripts in rice, the miRNA degradome transcripts identified in the six types of seeds were enriched in a total of 236 GO terms and 17 KEGG pathways (Table S3). When considering all annotated transcripts in this study as the background, the WT, *MIM164c*, and *OE164c* seeds, regardless of aging, showed different GO and KEGG enrichments for the unique target genes (Fig. 3B; Additional file 2: Fig. S1-S3). In the biological process GO category, only ‘SCF-dependent proteasomal ubiquitin-dependent protein catabolic process’ was collectively enriched by some degradome transcripts unique to artificially aged *OE164c* or WT seeds (Additional file 2: Fig. S1), and in the cellular component category, only ‘mitochondrion’ was collectively enriched by some degradome transcripts unique to unaged and aged WT seeds or unaged *MIM164c* seeds (Additional file 2: Fig. S3). In the KEGG pathways, only ‘ascorbate and aldarate metabolism’ was collectively enriched by some degradome transcripts unique to unaged *OE164c* or WT seeds, and ‘glycolysis/gluconeogenesis’ was collectively enriched by some degradome transcripts unique to unaged *MIM164c* or *OE164c* seeds (Fig. 3B). In addition, the degradome transcripts common to unaged and aged WT and *OE164c* seeds were mainly enriched in four GO terms, including ‘protein targeting to vacuole involved in ubiquitin-dependent protein catabolic process via the multivesicular body sorting pathway’ ‘protein serine/threonine phosphatase activity’ ‘zinc ion binding’ and ‘flavonoid biosynthetic process’ (Fig. 3C), and four KEGG pathways including ‘carotenoid biosynthesis’ ‘2-oxocarboxylic acid metabolism’ ‘plant hormone signal transduction’ and ‘plant-pathogen interaction’ (Fig. 3D). However, degradome transcripts common to unaged and aged WT and *MIM164c* seeds were enriched in none of the GO terms or KEGG pathways. The results showed that due to the differences in the expression levels of miR164c in seeds of different genotypes and whether they were artificially aged or not, the degradome transcripts were also different, and thus the functional clusters, GO and KEGG enrichments of target genes were different, which ultimately lead to differences in seed vigor or anti-aging ability.

Moreover, The subcellular localization pattern of some proteins sometimes matches the metabolic needs of a tissue [26]. In this study, based on the degradome transcripts, the subcellular distribution of 478 out of 1247 proteins encoded by miRNA target genes was successfully predicted (Table S4). These target proteins were mainly distributed in the nucleus, cell membrane, mitochondria, Golgi apparatus, ER and other organelles (Additional file 3: Fig. S4, Table S4). The subcellular distributions and proportions were different between the unaged and aged seeds of WT, *MIM164c* and *OE164c* genotypes. Among them, the targets of miR5075 were widely distributed in the nucleus, cytoskeleton, cytosol, endosome, extracellular region or secreted, plasma membrane, vacuole and mitochondria (Table S4), suggesting that miR5075 might play multiple roles in regulating seed vigor.

### miR164c and other miRNAs regulate seed vigor through their interactions with target genes as well as other functional genes

**Fig. 4:**
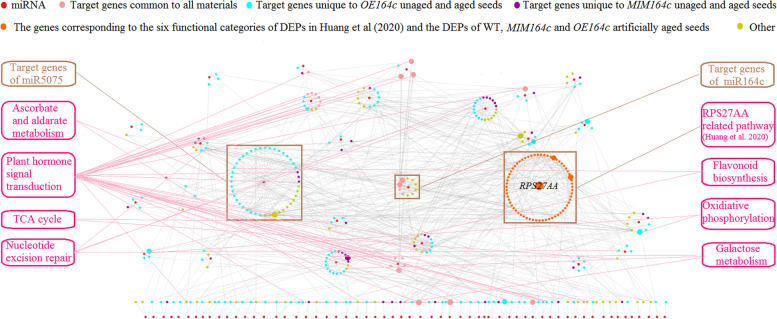
The miRNA-mediated gene interaction network regulating rice seed vigor. Big and small nodes represent direct and indirect interactions between miR164’s target genes and other genes, respectively

To further explore the molecular mechanism of seed vigor regulation by miRNAs, we analyzed the interactions among all target genes of miRNAs identified in the present study using the STRING database, an online database resource search tool for retrieving interacting genes, comprehensively covering relevant experimental and predicted gene interaction information. We also analyzed genes corresponding to the six metabolic functional categories of rice seed vigor related differentially expressed proteins (DEPs) reported in Huang et al. (2020) [11] as well as genes corresponding to DEPs among artificially aged WT, *MIM164c*, and *OE164c* seeds (1.3 < FC < 1/1.3). The results revealed an interaction network (Fig. 4) comprising 87 miRNA (family) genes, 298 target genes and 64 DEP-corresponding genes (Table S5); five of the miRNA target genes were identical to the DEP-corresponding genes. Among the miRNA target genes, 25 were common to all unaged and aged seeds; 158 were unique to unaged and aged *OE164c* seeds; 43 were unique to unaged and aged *MIM164c* seeds; and 72 represented other genes (i.e., target genes other than the above three types).

**Fig. 5:**
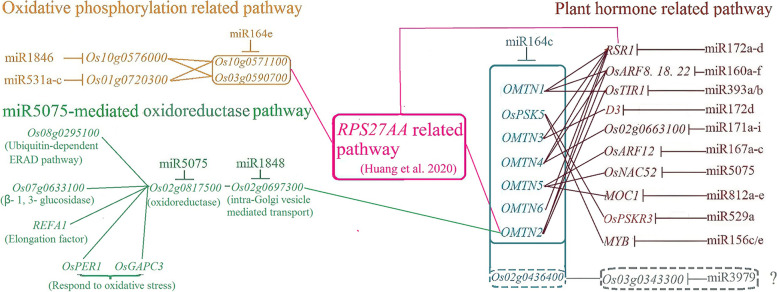
The simplified gene interaction network regulating rice seed vigor by directly interacting with miR164s’ target genes

In addition to the previously reported miR164c-guided *RPS27AA*-related pathway [11], the network contained at least other seven KEGG pathways: ‘ascorbate and aldarate metabolism’ ‘plant hormone signal transduction’ ‘galactose metabolism’ ‘nucleotide excision repair’ ‘TCA cycle’ ‘oxidiative phosphorylation’ and ‘flavonoid biosynthesis’ (Fig. 4), of which the *RPS27AA*-related pathway and three KEGG pathways that directly interact with the target genes of miR164s were simplified as Fig. 5. The results suggested that a miRNA-mediated integrative gene interaction network regulates seed vigor in rice. In the network, ‘ascorbate and aldarate metabolism’ was enriched by the miR5075 target gene *Os02g0817500* and the miR5821 target gene *Os01g0901300* (Fig. 3B, Table S5). It has been reported that the expression of genes related to ‘ascorbate and aldarate metabolism’ was down-regulated in artificially aged wheat (*Triticum aestivum* L.) seeds [27]. Abscisic acid (ABA) and auxin are reported to play a key role in regulating seed longevity and seed vigor [28]. Here the ‘plant hormone signal transduction’ pathway included 33 target genes of miRNAs, of which 12 directly interacted with the target genes of miR164c (Fig. 3D, Figs. 4 and 5). Among these 12 target genes, 1 target gene of a certain miRNA interacted with multiple target genes of miR164c and vice versa. The ‘galactose metabolism’ pathway was enriched by the miR5809 target gene *Os07g0209100* and *Os10g0492900,* and miR820 target gene *Os03g0255100* (Fig. 3B, Table S5). In a previous study on hybrid rice seeds, galactose and gluconic acid contents were significantly negatively correlated with the germination rate under different aging treatments [29]. The ‘TCA cycle’ pathway was enriched by the miR444a/d target gene *Os03g0773800* and miR2104 target gene *Os02g0595500* (Fig. 3B, Table S5). In the artificially aged seeds of oat (*Avena sativa* L.), Mao et al. [3] reported that the expression of some proteins related to the tricarboxylic acid (TCA) cycle is down-regulated, and the application of nitric oxide improves seed vigor by enhancing the mitochondrial TCA cycle and activating alternative pathways. The ‘nucleotide excision repair’ pathway included the miR2102 target gene *Os02g0633400*, the miR2104 target gene *Os05g0198700*, and the miR414 target genes *Os05g0592500* and *Os01g0779400* (Table S5). Ventura et al. [30] reported that the ‘nucleotide excision repair’ pathway is critical for ensuring genome stability and consequently enhancing seed vigor and improving the stress tolerance of germinating seeds. The miR5075 target gene *Os03g0819600* and miR5809 target gene *Os10g0379100* were enriched in the ‘flavonoid biosynthesis’ pathway (Fig. 3B, Table S5), which is reported to be related to antioxidant function [31].

Among all miRNAs, miR5075 showed the highest number of target genes (147) (Table S2), of which 44 were included in the network. Among these 44 target genes, 30 were unique to *OE164c* seeds, and only 3 were unique to *MIM164c* seeds. In the network, except for its involvement in the above mentioned KEGG pathway ‘ascorbate and aldarate metabolism’, miR5075 also indirectly participates in the *RPS27AA* and plant hormone related pathways, in which the miR1848 target gene *Os02g0697300* acts as a hub by bridging the gap between *Os02g0817500* and the miR164c target gene *OMTN2* (*TIL1*). Moreover, miR1848 potentially plays an important role in modulating the size and quality of rice seeds by regulating phytosterol and brassinosteroid (BR) biosynthesis through directing the mRNA cleavage of the obtusifoliol 14α-demethylase gene *OsCYP51G3* [32]. In addition, miR5075 likely also participates directly in the plant hormone related pathway through its target gene *NAC52*, which interacts with the miR164c target gene *OMTN5* (Fig. 5).

In addition, some of the miR164c target genes were also targeted by other members of the miR164 family, especially miR164e, which participated in the *RPS27AA* related pathway via two target genes, *Os10g0571100* and *Os03g0590700*. Both these genes also interacted with two oxidative phosphorylation related genes, *Os10g0576000* and *Os01g0720300*, which represent the target genes of miR1846 and miR531a–c, respectively. These results imply that other members of miR164 family are functionally redundant to miR164c (Fig. 5).

**Fig. 6:**
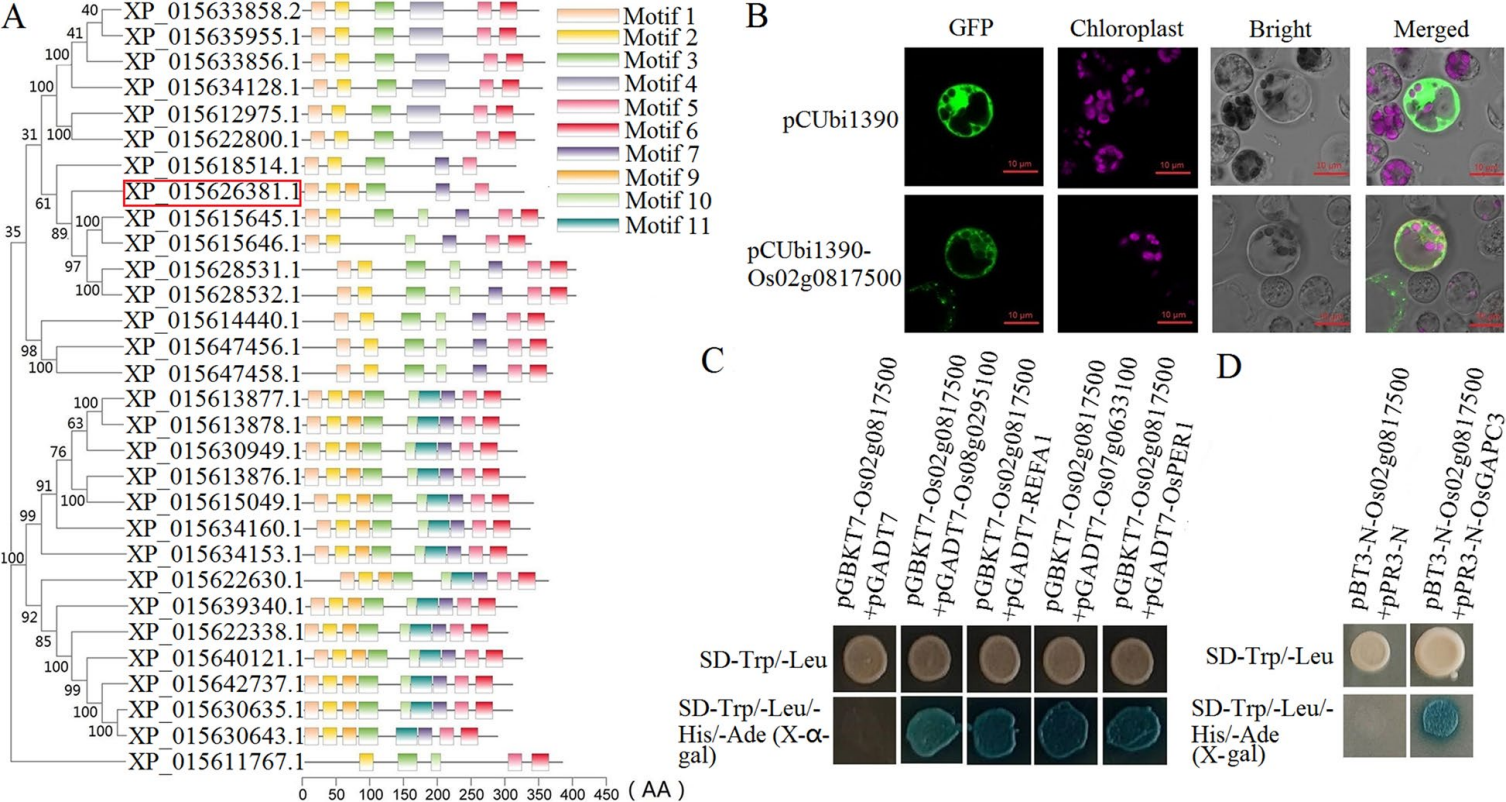
Phylogenetic analysis, subcellular localization and yeast two-hybrid (Y2H) assays of Os02g0817500. **A** Phylogenetic tree. XP_015626381.1, outlined with a red box represents Os02g0817500. **B** Subcellular localization of Os02g0817500, yellow-green indicated that Os02g0817500 expressed in the cytoplasm. **C** Nuclear system and (**D**) membrane system Y2H assays. The interaction partners of Os02g0817500 were identified by growing yeast on SD/−Leu/−Trp/−His/−Ade/X-α-gal and SD/−Leu/−Trp/−His/−Ade/X-gal plates. The pGADT7 and pPR3-N empty vectors were used as negative controls in the nuclear system and membrane system Y2H assays, respectively

Because *Os02g0817500* was the only miR5075 target gene enriched in the miR5075-mediated oxidoreductase pathway (i.e. the KEGG pathway ‘ascorbate and aldarate metabolism’) in the network, we conducted phylogenetic analysis to identify proteins homologous to *Os02g0817500*. The results showed that the homologs belonged to the Aldo/keto reductase (AKR) family (Fig. 6A). Plant AKRs have been shown to be effective in the detoxification of lipid peroxidation-derived reactive aldehydes [33]. The rice *OsAKR* genes are highly homologous to the stress-inducible aldo–keto reductase genes of *Medicago sativa* (*MsALR*), and *OsAKR*1 is involved in abiotic stress-related reactive aldehyde detoxification [34]. *AKR1* also improves seed longevity in tobacco and rice by detoxifying reactive cytotoxic compounds generated during aging [35]. *AKR4C14* (*OsAKR2*) in rice, like the dicot *AKR4Cs*, is also involved in the detoxification of reactive aldehydes [36].

Subcellular localization analysis indicated that Os02g0817500 protein mainly functions in the cytoplasm (Fig. 6B).

In the Y2H assay, proteins encoded by five seed vigor-related genes, *Os08g0295100*, *Os07g0633100*, *REFA1*, *OsPER1* and *OsGAPC3*, interacted with the miR5075 target protein Os02g0817500 (Fig. 6C).

### Verification of the function of seed vigor-related miRNAs and target genes

**Fig. 7:**
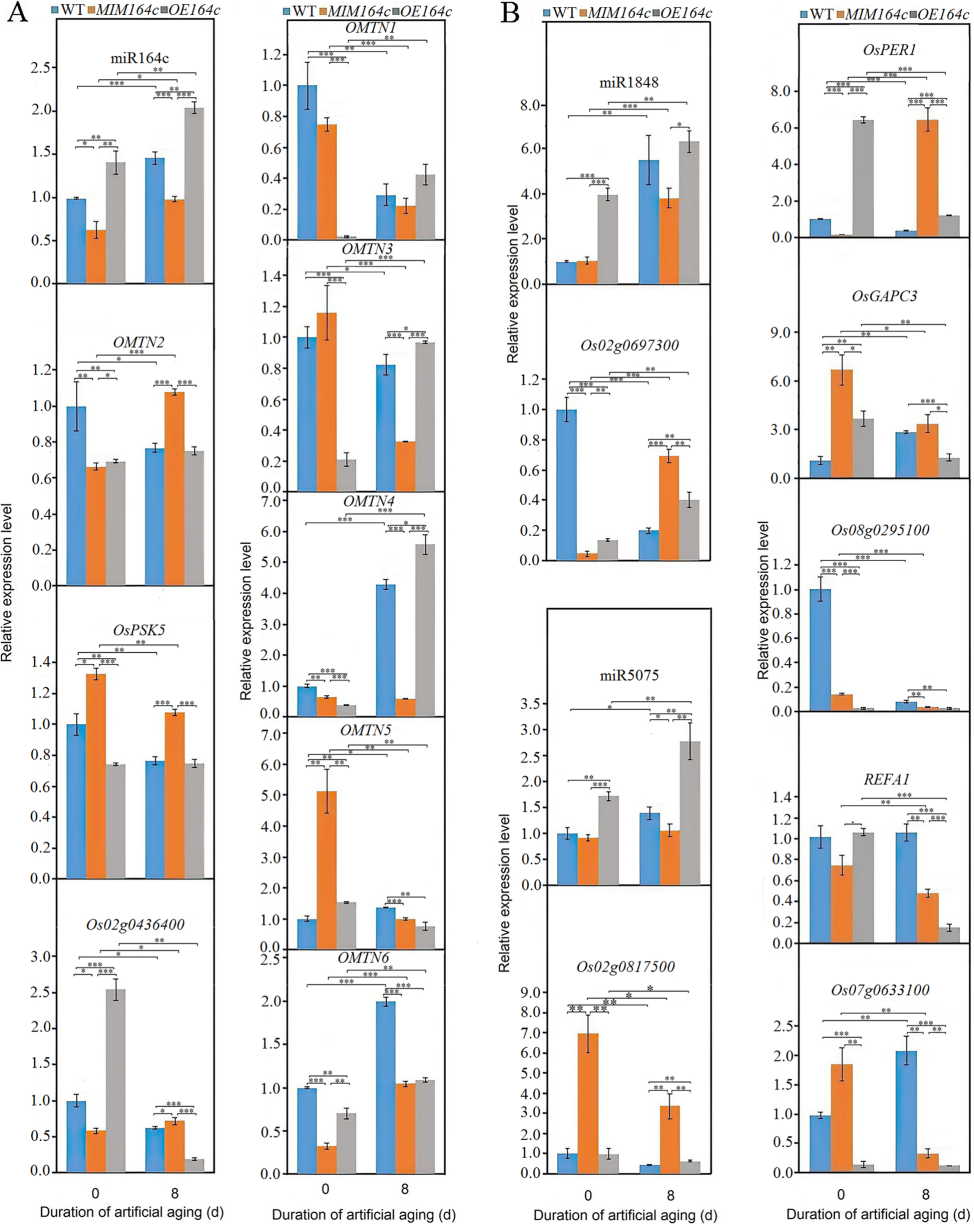
RT-qPCR analysis of the expression of miR164c and target genes, and miR5075-mediated oxidoreductase pathway related genes. (**A**, **B**) Expression levels of miR164c and its target genes *OsPSK5*, *Os02g0436400* and *OMTN1–6* (**A**) and miR5075-mediated oxidoreductase pathway related genes including miR1848 and target gene *Os02g097300*, miR5075 and target gene *Os02g0871500*, and the interacting genes *OsPER1*, *OsGAPC3*, *Os08g0295100*, *REFA1* and *Os07g0633100* (**B**). Data represent mean ± SD (*n* = 3). Significant differences among different samples were determined using Student’s *t*-test (*P < 0.05, **P < 0.01, ***P < 0.001). The expression level of the gene of interest in unaged WT seeds was set as 1

To verify whether other miRNAs and miR164c regulate seed vigor through their interaction with target genes, we performed RT-qPCR analyses and characterized the relationship between rice seed vigor and the expression of miRNA target genes in the network. *OMTN2* and *OsPSK5*, two target genes of miR164c, were linked with each other in the network through multiple regulatory pathways (Fig. 5). Although the seed vigor of all three genotypes decreased with artificial aging, resulting in significant differences among the genotypes, the *MIM164c* line showed the least reduction in seed vigor (Fig. 1), which is highly consistent with our previous reports [10, 11]. Expression levels of *OMTN2* and *OsPSK5* were significantly higher in *MIM164c* seeds than in WT and *OE164c* seeds, as expected, which was opposite to that of miR164c. However, no significant difference in the expression level of *OMTN2* or *OsPSK5* was observed between WT and *OE164c* seeds, which is puzzling and requires further investigation (Fig. 7A). Expression levels of miR5075 and miR1848 were consistent with that of miR164c; artificial aging enhanced the expression levels of miR5075 and miR1848 in both unaged and aged seeds, and their resultant expression levels were the lowest in *MIM164c* seeds and highest in *OE164c* seeds (Fig. 7B). Differences in the expression level of the miR1848 target gene *Os02g0697300* among WT, *MIM164c*, and *OE164c* seeds (aged and unaged) were similar to those in the expression level of *OMTN2*. The miR5075 target gene *Os02g0817500* was expressed to the highest levels in *MIM164c* seeds, regardless of aging, which was negatively correlated with the expression level of miR5075.

*Os02g0436400* is a new target gene of miR164c identified in the present study, and is worthy of attention since its expression level was strongly negatively correlated with that of miR164c in aged WT, *MIM164c*, and *OE164c* seeds (Fig. 7A), and positively correlated with seed vigor. In the interaction network, *Os02g0436400* showed interaction with the miR3979 target gene *Os03g0343300* (Fig. 5); however, the biological functions of *Os02g0436400* and *Os03g0343300* have not yet been reported.

In the miR5075-mediated oxidoreductase pathway, the target gene *Os02g0697300* of miR1848 interacted with the target gene *Os02g0817500* of miR5075 (Fig. 5). Although Y2H analysis failed to identify the interaction between *Os02g0697300* and *Os02g0817500* (Fig. 6C), the expression levels of *Os02g0697300* and *OsPER1,* one of the interacting genes of *Os02g0817500*, were consistent with that of *Os02g0817500* in the aged WT, *MIM164c* and *OE164c* seeds (Fig. 7B). This suggested that *Os02g0697300* and *OsPER1* may play a major role in the miR5075-mediated oxidoreductase pathway to regulate seed vigor or anti-aging ability.

**Fig. 8:**
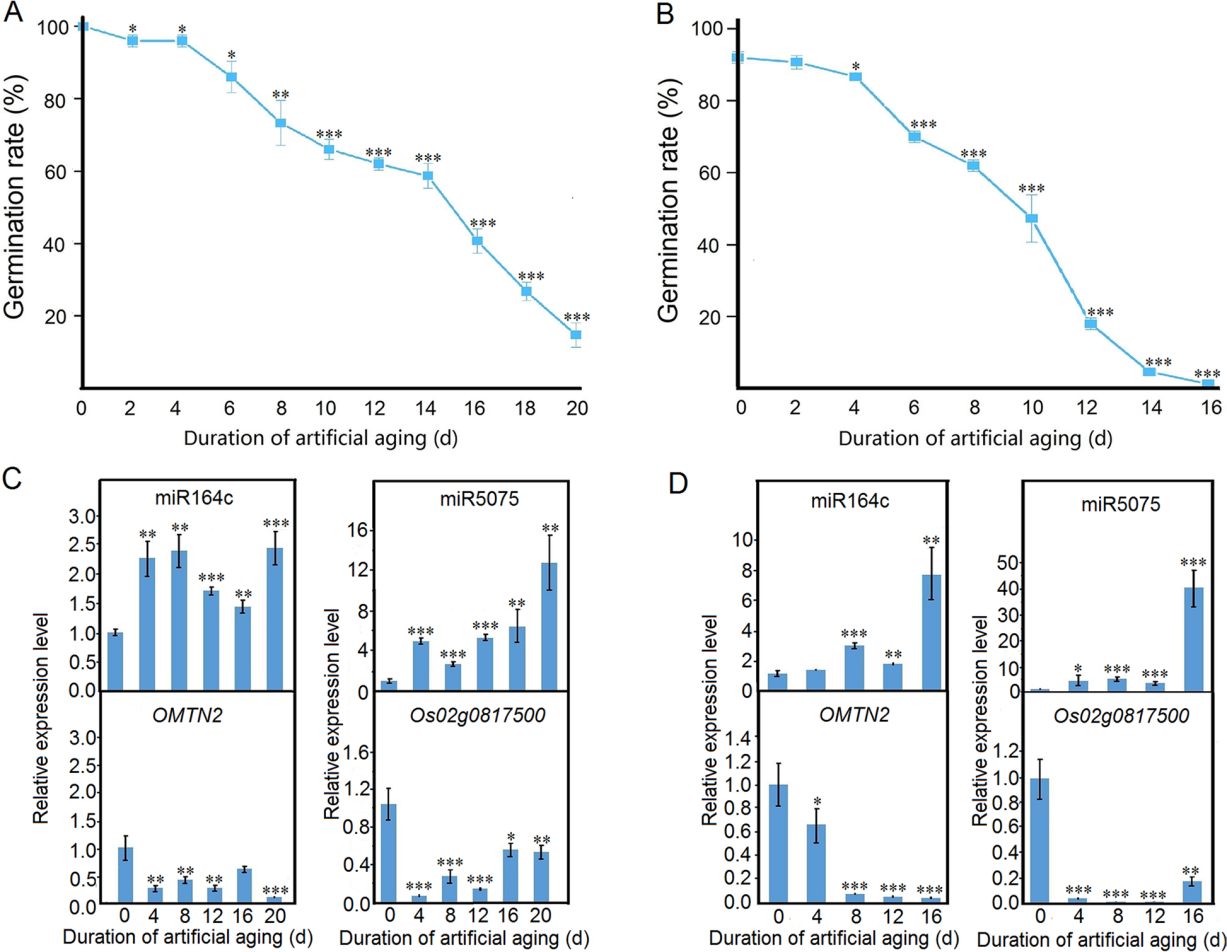
Artificial aging-induced changes in seed germination rate and RT-qPCR analysis of the gene expression levels in rice cultivars ‘Kasalath’ and ‘Nipponbare’. (**A**–**D**) Seed germination rate and seed-specific expression levels of miR164c, miR5075 and the respective target genes *OMTN*2 and *Os02g0817500* in Kasalath (**A**, **C**) and Nipponbare (**B**, **D**). Data represent mean ± SD (*n* = 3). Significant differences between the unaged and seeds of each cultivar with different degrees of aging were determined using Student’s *t*-test (*P < 0.05, **P < 0.01, ***P < 0.001). In (**C**, **D**), the expression level of the gene of interest in unaged WT seeds was set as 1

Moreover, in ‘Nipponbare’ and ‘Kasalath’ seeds artificially aged for 0, 4, 8, 12, 16, and 20 days, the declining seed vigor-related changes in the expression of miR5075 and its target gene *Os02g0817500* were highly consistent with those of miR164c and the target gene *OMTN2*, respectively (Fig. 8).

**Fig. 9:**
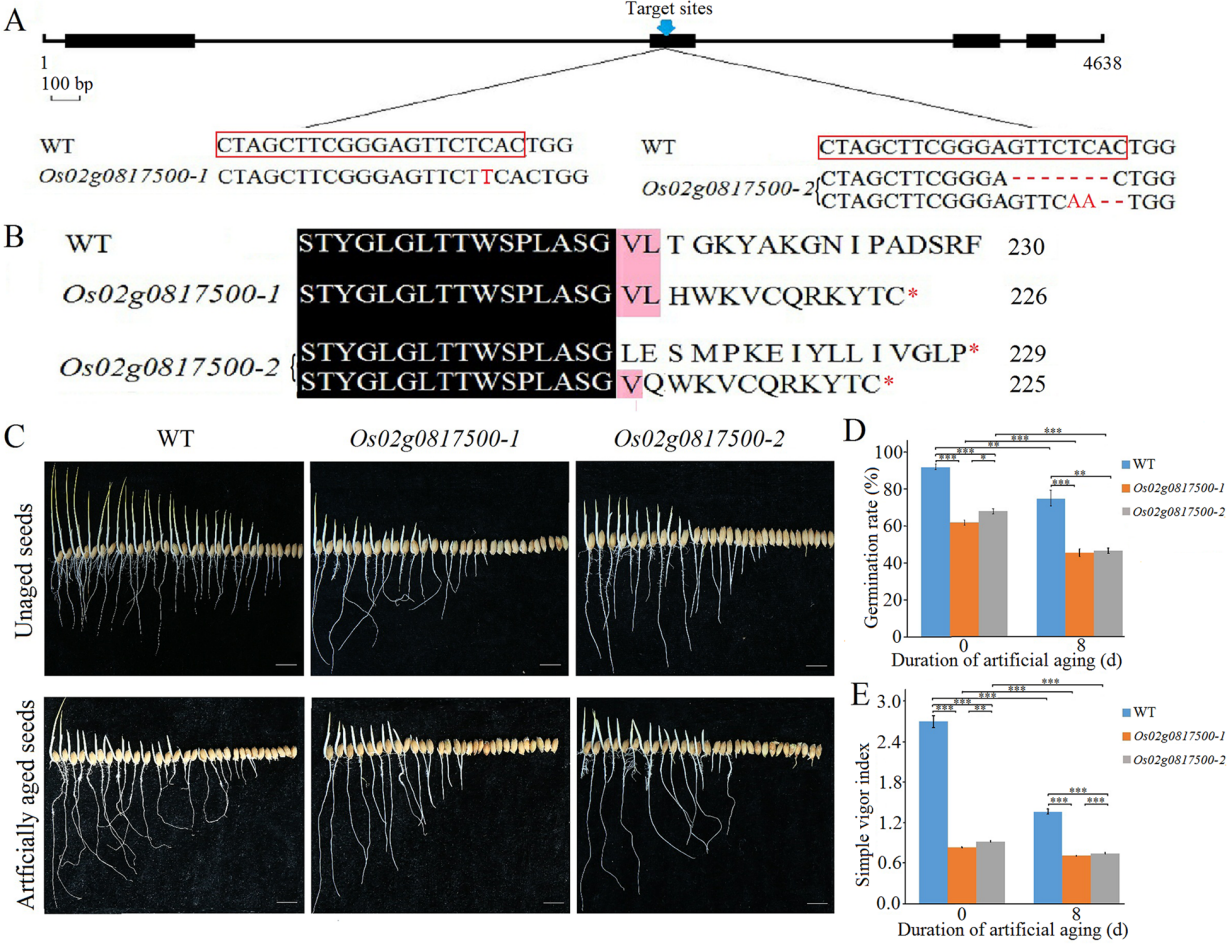
Effect of *Os02g0817500* knockout on the seed vigor of rice ‘Nipponbare’. **A** Target sites in the wild type (WT) are marked in red box. Inserted nucleotide is indicated with red uppercase letter and red dashes represent the deleted bases. The original data can be viewed from Additional file 4: Fig. S5. **B** Analysis of the Os02g0817500 protein sequence in the wild-type (WT) and *Os02g0817500*-knockout lines. **C** Photographs of germinated seeds before and after artificial aging of *Os02g0817500*-knockout lines and ‘Nipponbare’ (WT) after 5 days after germination. (**D**, **E**) Comparison of germination rate (**D**) and simple vigor index (**E**) between *Os02g0817500*-knockout lines and ‘Nipponbare’. Bar = 1 cm. Each column presents the means ± standard deviations of three biological replicates. *P < 0.05, **P < 0.01 and ***P < 0.001 compared with the WT by Student’s t-test

Two knockout mutants (a homozygous line *Os02g0817500–1* and a bi-allelic line *Os02g0817500–2*) were generated in the background of ‘Nipponbare’ through the CRISPR/Cas9 system. The *Os02g0817500*–*1* contained a “T” insertion, and the *Os02g0817500*–*2* a “7 bp (GTTCTCA)” deletion and a 2 bp substitution (“TC” substuted by “AA”) plus “2 bp (AC)” deletion in the second exon of *Os02g0817500*, respectively (Fig. 9A and Additional file 4: Fig. S5). Based on these nucleotide sequences, the amino acid sequence of *Os02g0817500* was predicted to contain only 226 and 225 / 229 amino acids in *Os02g0817500*–*1* and *Os02g0817500–2* mutants, respectively, which were caused by premature termination (Fig. 9B). This result indicates that the mutants lacked *Os02g0817500* gene. Germination assays showed that the germination rate and simple vigor index of two transgenic (*Os02g0817500*–*1* and *Os02g0817500*–*2*, T_1_ generation) seeds before and after artificial aging were significantly reduced compared with that of Nipponbare (Fig. 9C-E), suggesting the disruptions of *Os02g0817500* gene significantly decreased seed vigor and anti-aging ability.

**Fig. 10:**
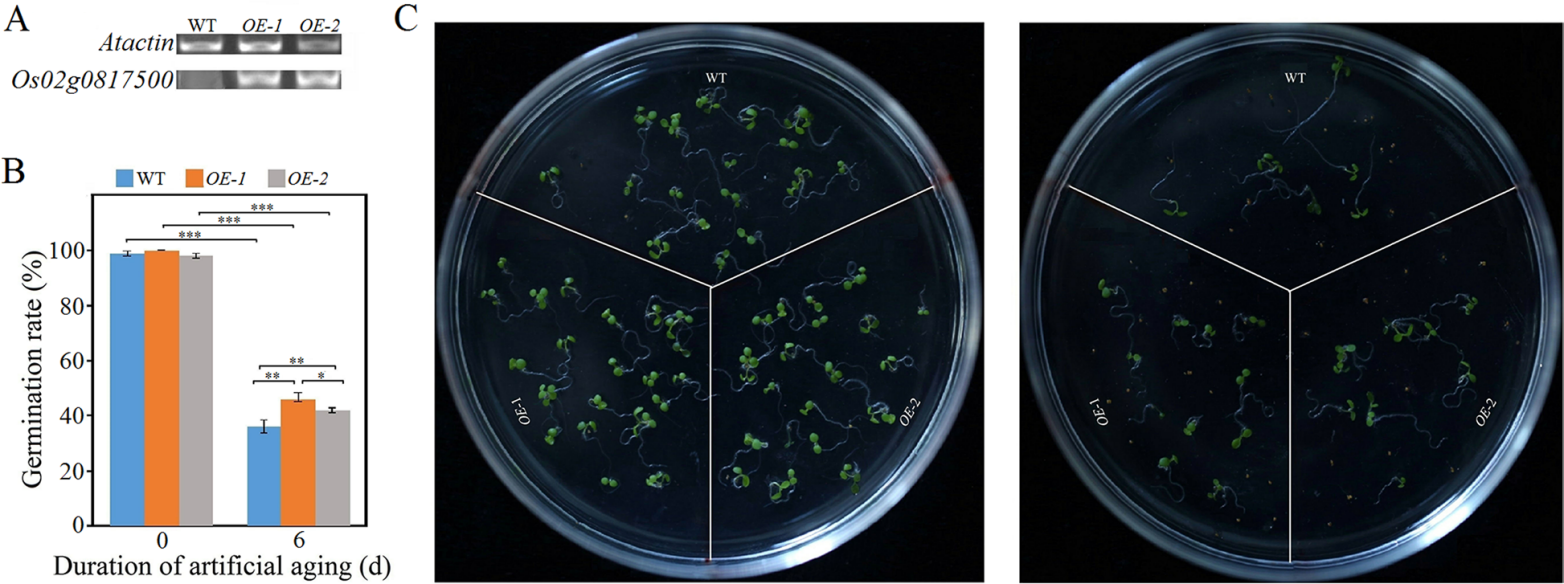
Effect of aging on the germination performance of *Arabidopsis* (Col- WT) and *Os02g0817500* ectopic expression lines ‘*OE-*1’ and ‘*OE-*2’. **A** Semi-quantitative RT-PCR analysis of the expression of *Os02g0817500* in *Arabidopsis* leaves. *Atactin* was used as a positive control. The original data can be viewed from Additional file 5: Fig. S6. **B** Germination rates of seeds before and after artificial aging. Data represent mean ± SD (*n* = 3). Significant differences between WT and transgenic samples were determined using Student’s *t*-test (*P < 0.05, **P < 0.01, ***P < 0.001). **C** Phenotypes of 5-day-old seedlings produced from unaged seeds (Left) and 10-day-old seedlings produced from artificially aged seeds (Right). Seedlings were grown on the half-strength Murashige and Skoog (MS) medium

In addition, we generated two *Arabidopsis* transgenic lines (*OE*-1 and *OE*-2) ectopically expressing *Os02g0817500* (Fig. 10A and Additional file 5: Fig. S6). Before artificial aging, the *OE*-1, *OE*-2 and WT seeds showed no significant differences in their viability; however, after artificial aging, seeds of both ‘*OE*-1’ and ‘*OE*-2’ lines showed higher germination rates and grew more rapidly than those of the WT (Fig. 10B and C).

Additionally, 12 target genes of 10 miRNAs (family), which directly interacted with the target genes of miR164c, were involved in the plant hormone related pathway in the network (Figs. 4 and 5). The numbers of degradome transcripts of these target genes in unaged and artificially aged seeds of WT, *MIM164c*, and *OE164c* genotypes are listed in Table 3. The number of degradome transcripts of most target genes increased with the aging of seeds of all genotypes. However, except for miR172d, miR812a–e, miR529a, and miR156c/e, whose target gene degradome transcripts were either undetectable or too low in WT and *MIM164c*, the numbers of degradome transcripts of the target genes of the other six miRNAs were much higher in aged WT and *OE164c* seeds than in aged *MIM164c* seeds. In *MIM164c* seeds, degradome transcripts of the target genes of miR160a–f (*OsARF18* and *OsARF22*) and miR167a–c (*OsARF12*) were fewer in aged seeds than in unaged seeds. Next, to investigate the effect of miRNAs on the expression of target genes involved in the plant hormone related pathway, we examined the expression levels of eight miRNAs and eight corresponding target genes by RT-qPCR. However, it was puzzling that only the expression level of miR393a and its target gene *OsTIR1* showed a negative correlation in unaged and aged WT, *MIM164c*, and *OE164c* seeds; the other miRNAs and corresponding target genes showed an irregular expression correlation in the unaged and aged seeds of all genotypes (Additional file 6: Figue S7). Whether this result was caused by the complexity of the regulatory action of these plant hormone related genes or technical errors remains to be verified.

**Fig. 11:**
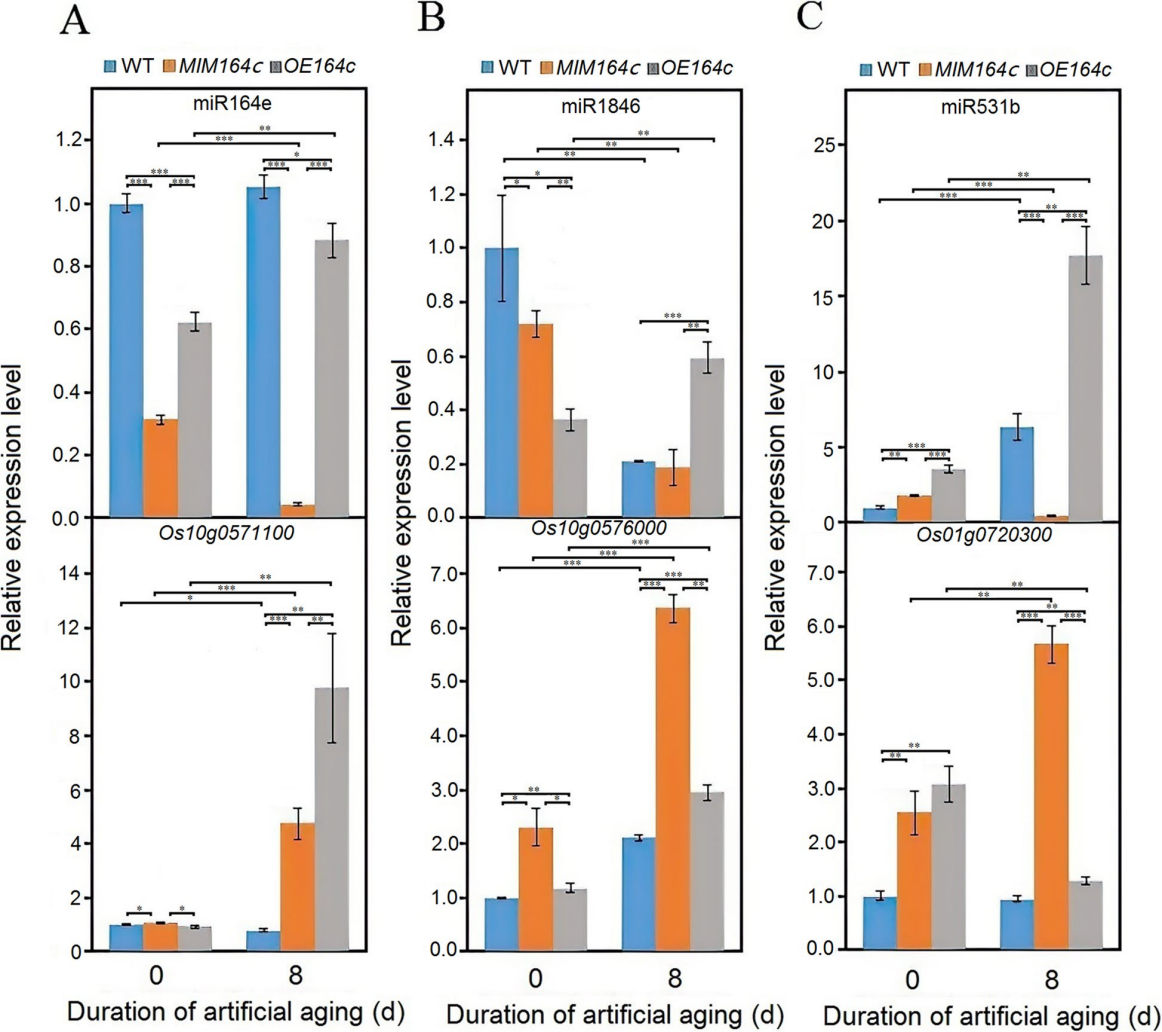
RT-qPCR analysis of the expression of genes involved in the miR164e-mediated oxidative phosphorylation related pathway. (**A**) miR164e and the target gene *Os10g0571100*; (**B**) miR1846 and the target gene *Os10g0576000*; (**C**) miR531b and the target gene *Os01g0720300*. Data represent mean ± SD (*n* = 3). Significant differences among different samples were determined using Student’s *t*-test (*P < 0.05, **P < 0.01, ***P < 0.001). The expression level of the gene of interest in unaged WT seeds was set as 1

Moreover, among the seeds of three different genotypes, the expression level of miR164e, like that of miR164c, was the lowest in *MIM164c* seeds, regardless of aging, suggesting that miR164c and miR164e are functionally redundant. Analysis of degradome data revealed two genes as targets of miR164e, *Os10g0571100* and *Os03g0590700*. RT-qPCR analysis indicated that the expression level of miR164e was negatively correlated with that of *Os10g0571100* in WT and *MIM164c* seeds, but not in *OE164c* seeds, both before and after aging (Fig. 11A); the expression of *Os03g0590700* was not detectable in any genotype. In addition, the expression level of *Os10g0571100* was not consistent with that of its two interacting genes *Os10g0576000* and *Os01g0720300* in all seeds, regardless of aging (Fig. 11B and C). Both *Os10g0571100* and *Os03g0590700* also showed interaction with the functional genes in the *RP*27*AA* related pathway (Fig. 5); however, how miR164e plays a role in this interaction network remains unclear.

## Discussion

In plants, miRNAs are involved in all aspects of growth and development. We previously showed that the aging process of rice seeds is accompanied by changes in the expression levels of some miRNAs, especially miR164c, which is negatively correlated with seed vigor [10, 11]. This result was further verified in the present study (Fig. 1). The miRNA-mediated mRNA degradation is a critical factor in determining mRNA abundance, allowing plants to rapidly regulate gene expression in response to various stresses [37]. However, little is known about the role of mRNA degradation during seed aging. Degradome sequencing can provide huge amounts of data on RNA degradation to confirm the miRNA-mediated cleavage of target transcripts and to identify new target genes [38]. To explore whether the miR164c-guided regulation of seed vigor involves other miRNAs and their target genes, we performed degradome sequencing analysis of WT, *MIM164c*, and *OE164c* rice seeds before and after aging. A total of 1247 target transcripts potentially cleaved by 421 miRNAs were identified. Additionally, regardless of aging, the number of miRNAs and degradome transcripts in *OE164c* seeds was higher than that in WT and *MIM164c* seeds (Fig. 2), and the numbers of degradome transcripts of miR164c in *OE164c* seeds were also greater than that in WT and *MIM164c* seeds (Table 2). These results suggest that miR164c expression-related differences in degradome transcripts among seeds of different genotypes may be closely related to seed vigor or anti-aging ability.

### Degradation of the target transcripts of miR164c and other miRNAs potentially contributes to seed vigor decline

In wheat, a complex miRNA-mediated regulatory network regulates the response to *Fusarium graminearum* infection [39]. In rice seedlings at the three-leaf stage, the miRNA target genes are mainly enriched in the transcription factor activity, response to endogenous stimulus and other GO functions [40]. In mature rice seeds (current study), although the target genes of miRNAs were enriched in the transcription factor activity, mitochondrial genome maintenance, ATP binding and other GO functions, none were enriched in the response to endogenous stimulus (Table S3). This suggests that the functional enrichment of target genes identified in different tissues and at different growth stages of rice is not identical, and the differences may be attributed to the regulatory control of miRNAs, which shows variation in time and space. Seed vigor may also be associated with specific target genes of miRNAs. Target genes unique to the unaged seeds of the WT or *OE164c* line were enriched in the KEGG pathway ‘ascorbate and aldarate metabolism’ (Fig. 3B), and target genes common to unaged and aged WT and *OE164c* seeds were enriched in four GO functions and four KEGG pathways, but no target genes common to unaged and aged WT and *MIM164c* seeds were enriched in any of the abovementioned GO functions or KEGG pathways (Fig. 3C and D). The KEGG pathway ‘flavonoid biosynthesis’ is reported to be related to antioxidant function [31]. In addition, some miRNA target genes common to WT and *OE164c* seeds, but not to *MIM164c* seeds, were enriched in ‘protein serine/threonine phosphatase activity’ and other GO functions (Fig. 3C); on the other hand, some target genes unique to unaged *OE164c* seeds were enriched in ‘ER to Golgi vesicle-mediated transport’, and some target genes unique to aged *OE164c* seeds were enriched in ‘response to abscisic acid’ and ‘ethylene-activated signal pathway’ (Additional file 2: Fig. S1). Together, all of these functions are reported to be involved in seed vigor regulation and the plant hormone signaling pathway [41, 42]. Degradation of these function-related target transcripts by miRNAs may lead to hormone signal transduction dysfunction in *OE164c* seeds and reduction in seed vigor. Target transcripts related to energy metabolism such as the TCA cycle were degraded in *OE164c* seeds to a greater extent than in WT seeds; however, no degradation products of the same target transcripts were detected in *MIM164c* seeds (Fig. 3B). This result is consistent with the higher expression levels of genes related to energy metabolism in *MIM164c* seeds than in WT and *OE164c* seeds [11]. Moreover, subcellular localization prediction showed that most of the proteins encoded by miRNA target genes corresponding to the degradome transcripts in the Golgi apparatus and ER were unique to unaged and aged *OE164c* seeds (Additional file 3: Fig. S4). Protein content is closely related to seed vigor [43]. The Golgi apparatus and ER are involved in protein processing, which may decrease the content of mature proteins and thus affect seed vigor. The above results suggest that the low anti-aging ability of *OE164c* seeds could potentially be attributed to the degradation of a greater number of unique target transcripts by miRNAs in *OE164c* seeds than in WT and *MIM164c* seeds.

### miR5075 and its target gene ***Os02g0817500*** potentially play pivotal roles in the miR164c-guided interaction network to regulate seed vigor

Degradome sequencing indicated that the number of miR5075 degradome transcripts in aged *OE164c* seeds was approximately twice as high as that in the other five seed samples (Table S2). In the miR164c-guided interaction network, the miR5075 target gene *Os02g0817500* showed direct interactions with multiple functional genes and indirect interactions with the miR164c target gene *TIL1* (*OMTN2*) (Figs. 4 and 5). By Y2H analysis, the proteins encoded by five genes (*Os08g0295100*, *Os07g0633100*, *REFA1*, *OsPER1* and *OsGAPC3*) were identified to interact with Os02g0817500 (Fig. 6C and D). Among them, *OsPER1* and miR1848 target gene *Os02g0697300* were noteworthy, as their corresponding gene expression level in aged WT, *MIM164c*, and *OE164c* seeds was highly consistent with the expression level of *Os02g0817500* (Fig. 7B). PER1 is a seed-specific antioxidant in many plants that uses cysteine residues to scavenge ROS, ectopic expression of *Nelumbo nucifera* Gaertn (*NnPER1*) in *Arabidopsis* enhances the seed germination rate after controlled deterioration treatment, and improves the tolerance to high temperature and ABA, indicating that *NnPER1* participates in the thermotolerance and ABA signaling pathway [44]. It is recently reported that overexpression and knockout of *PER1A* enhances and decreases rice seed vigor, respectively [45]. Meanwhile, *Os08g0295100* is related to the ubiquitin-dependent endoplasmic reticulum-associated degradation (ERAD) pathway, which results in the accumulation of misfolded proteins in the ER, leading to further cell damage and accelerating the loss of seed vigor [46]. *Os07g0633100* is similar to the endo-1,3-β-glucosidase gene. β-Glucosidases (βGlus) are multifunctional enzymes that affect plant growth and their adaptation to the environment by controlling processes such as phytohormone activation, plant defense, cell wall oligosaccharide catabolism and cell wall lignifications [47]. *Os4BGlu14*, a rice β-glucosidase gene, negatively affects seed longevity during accelerated aging [48]. *REFA1* codes a translation elongation factor homologous to the eukaryotic translation elongation factor 1 α (eEF1A)-like protein, which is involved in programmed cell death (PCD) and acts as a link to defense responses in rice [49]. Seed aging also involves the process of PCD, which suggests that *REFA1* is involved in regulating the anti-aging ability of seeds. ROS are an important factor affecting seed deterioration, and their association with reduction in seed longevity and viability has been widely recognized [50]. *GAPDH* exhibits multifaceted functions in addition to its role in the glycolytic pathway [51]. In higher plants, GAPDH exists as three distinct isoforms, which exhibit cell-specific compartmentalization and are encoded by distinct nuclear genes. GAPDH is related to oxidative stress and PCD, and the *GAPDH* gene is up-regulated during seed aging [52]. Cytosolic GAPDH (GAPC) catalyzes the oxidative phosphorylation of glyceraldehyde-3-phosphate into 1,3-bisphosphoglycerate by converting NAD^+^ into the high energy electron carrier NADH [53]. The *OsGAPC*3 gene is induced most significantly by salt stress, and transgenic rice plants overexpressing *OsGAPC*3 show enhanced salt stress tolerance and increased hydrogen peroxide scavenging activity [54]. This suggests that *OsGAPCs* are involved in the regulation of seed vigor.

The expression level of *Os02g0817500* and that of its interacting genes differed significantly among WT, *MIM164c*, and *OE164c* seeds (Fig. 7B), suggesting that the expression of *Os02g0817500* and its interacting genes are affected by the differential expression of miR164c. Especially, the expression level of *Os02g0817500* was the lowest in *OE164c* seeds and the degradome transcripts of this gene were only detectable in *OE164c* seeds (Table S2). RT-qPCR analyses demonstrated that the expression level of miR5075 was negatively correlated with seed vigor, consistent with the correlation between miR164c expression level and seed vigor (Fig. 7). In ‘Kasalath’ and ‘Nipponbare’ seeds with different degrees of aging, the expression level of miR5075 target gene *Os02g0817500* was positively correlated with seed vigor (Fig. 8). Moreover, the knockout mutant seeds of *Os02g0817500* decreased seed vigor and anti-aging ability significantly compared with that of ‘Nipponbare’ (WT) (Fig. 9C-E); and *Arabidopsis* seeds ectopically expressing *Os02g0817500* showed greater anti-aging ability than WT seeds (Fig. 10). These suggest that miR5075 and its target gene *Os02g0817500* play important roles in the miR164c-guided interaction network to regulate seed vigor.

Moreover, it is also likely that miR5075 directly participates in the plant hormone related pathway in the network through the interaction of its target gene *OsNAC52* with the miR164c target gene *OMTN5* (Fig. 5). Both *OMTN5* and *OsNAC52* belong to the *NAC* gene family. The *OsNAC*52 gene functions as an important transcriptional activator of ABA-inducible genes, and therefore could be used to improve the abiotic stress tolerance of plants [55]. However, in the present study, the expression levels of *OMTN5* and *OsNAC52* were not consistent in WT, *MIM164c*, and *OE164c* seeds, regardless of aging, and did not show a negative correlation with the expression levels of the corresponding miRNAs, miR164c and miR5075, respectively. Further investigation is needed to understand whether and how miR5075 regulates the vigor or anti-aging ability of seeds through its target gene *OsNAC52*.

### miR164c-guided seed vigor regulatory network includes plant hormone related pathways and the functionally redundant miR164 family members

In the network, 12 target genes of 10 miRNAs were involved in the hormone related pathway by directly interacting with one or more miR164c target genes (Fig. 5). Among the miR164c target genes, *TIL1* (*OMTN2*) and *OsPSK*5 also acted as key hub genes in the *RPS*27*AA* related pathway to regulate rice seed vigor [11]. All of these target genes are implicated in the regulation of plant growth and development and stress resistance. For example, Rice Starch Regulator1 (RSR1), an APETALA2 (AP2)/ethylene-responsive element binding protein (EREBP) family transcription factor, negatively regulates endosperm starch biosynthesis and affects the starch quality and physicochemical characteristics of seeds by modulating the expression of starch biosynthesis genes [56]. The negative regulation of *OsARF18* expression by OsmiR160 affects rice growth and development via auxin signaling [57]. Additionally, miR167 regulates the expression of *OsARF6*, *OsARF*12, *OsARF17*, and *OsARF25* to contribute to the normal growth and development of rice [15]. Nitrogen fertilizer-induced OsmiR393 accumulation reduces the expression of *OsTIR1* and *OsAFB2*, which alleviates sensitivity to auxin in axillary buds and stabilizes the OsIAA6 protein, thereby promoting rice tillering [58]. OsDWARF3 (OsD3) is required for the strigolactone (SL) and karrikin signal-induced degradation of OsSMAX1, which is necessary for the inhibition of rice mesocotyl elongation in the dark [59]. The *MONOCULM1* (*MOC*1) gene is a key factor that controls the formation of rice tiller buds [60]. The miR529 target gene *OsPSKR3* encodes a candidate PSK receptor, and its homolog OsPSKR1 confers resistance to bacterial leaf streak by activating the expression of pathogenesis-related (*PR*) genes involved in the salicylic acid (SA) pathway in rice [61].

In the present study, in WT, *MIM164c* and *OE164c* seeds, the expression levels of miRNAs and corresponding target genes involved in the plant hormone related pathway, except miR393 and its target gene *OsTIR1*, did not show a negative or positive correlation with seed vigor before and after aging (Fig. 1, Additional file 6: Fig. S7). The TPB value of almost all degradome transcripts in aged WT and *OE164c* seeds were greater than those of target transcripts in *MIM164c* seeds, suggesting that the target transcripts are more easily degraded in aged WT and *OE164c* seeds than in aged *MIM164c* seeds, which potentially contributes to the lower anti-aging ability of WT and *OE164c* seeds compared with that of *MIM164c* seeds. Given the interactions of multiple miRNAs with the corresponding target genes and with multiple miR164c target genes involved in the plant hormone related pathway as well as the complex crosstalk among different plant hormone signals affecting plant processes, how these genes participate in the regulation of seed vigor or anti-aging ability requires further investigation.

MiR164c and other members of the miR164 family potentially act redundantly to regulate seed vigor or anti-aging ability. Degradome sequencing revealed that some target genes of miR164c were also targeted by other members of the miR164 family (Table S2). STRING database analysis revealed *Os10g0571100* and *Os03g0590700* as two unique targets of miR164e, which interacted with the target genes of miR1846 (*Os10g0576000*) and miR531a–c (*Os10g0576000* and *Os01g0720300*) and were associated with the oxidative phosphorylation related pathway. On the other hand, miR164e likely participates in the *RPS*27*AA* related pathway via *Os10g0571100* and *Os03g0590700* (Fig. 5).

## Conclusion

In conclusion, through degradome sequencing and STRING database analysis, an integrative miRNA-mediated gene interaction network regulating rice seed vigor was uncovered, which contained the previously reported *RPS27AA* related pathway [11] and at least three new pathways, i.e., the miR5075-mediated oxidoreductase related pathway, the plant hormone related pathway and other miR164 family members such as miR164e functionally redundant to miR164c related pathway. Although the mechanism of interaction among the genes in the network needs to be further elucidated, the results provide a new perspective on the molecular mechanism underlying seed vigor regulation.

## Methods

### Plant materials

Seeds of the wild-type rice (*Oryza sativa* L.) cultivars ‘Kasalath’ (an important model material for *indica* rice) and ‘Nipponbare’ (an important model material for *japonica* rice) were obtained from the Plant Development and Molecular Laboratory of Hunan Normal University, China. Two rice cultivars were authenticated by the co-author, Professor Mengliang Xu. The development and identification of the miR164c-silenced line ‘L13–1–2-1’ (*MIM164c*) and miR164c overexpression line ‘L4–1–3-1’ (*OE164c*), harboring the genes of interest under the control of the rice ubiquitin promoter in ‘Kasalath’ background, have been described previously [10]. The *MIM164c* and *OE164c* seeds used in this study were in the T_6_ generation.

The *Os02g0817500* mutants *Os02g0817500–1* and *Os02g0817500–2* (Nipponbare background) were generated by CRISPR-Cas9 system [62]. All transgenic plants were identified by hygromycin gene amplification. All DNA constructs and PCR products were confirmed by sequencing (Tsingke Biotech, Beijing). Specific primers were designed to confirm the mutation positions in each CRISPR/Cas9-positive transgenic line (Table S1). The results of sequencing of two *Os02g0817500*- knockout lines in ‘Nipponbare’ background were shown in Additional file 4: Fig. S5.

To generate transgenic *Arabidopsis* lines ectopically expressing *Os02g0817500*, the cDNA of *Os02g0817500* (without the stop codon) was cloned into the pCUbi1390 vector. After confirmed by sequencing (Tsingke Biotech, Beijing), the resultant vector was electroporated into *Agrobacterium tumefaciens* strain GV3101, which was used to transform *Arabidopsis* (Columbia-0 ecotype, Col-0) using the floral dip method [63]. The candidate transgenic seeds were germinated on medium containing 30 mg/L hygromycin to select transgenic plants. The identity of transgenic lines was confirmed by examining the expression of *Os02g0817500* by the semi-quantitative reverse transcription polymerase chain reaction (semi-quantitative RT-PCR), as described by Huang et al. [64].

All primers are listed in Table S1. At least one voucher specimen for each of the above materials has been deposited in the Plant Development and Molecular Laboratory of Hunan Normal University, China.

### Seed germination test

Fifty rice seeds surface-sterilized with 3% NaClO were randomly arranged in a filter paper-lined Petri dish (90-mm diameter). Then, 10 mL of pure water (resistance, 18.2 MΩ•cm^− 1^ at 25 °C; total organic carbon (TOC), < 10 ppb) was dispensed onto the filter paper, and the Petri dish was incubated at 28 °C for 7 days in an environmentally controlled growth chamber to germinate seeds. Seeds were considered to have germinated when the length of the radicle was greater than that of the seed, and the length of the plumule was greater than half that of the seed. Seed germination tests were performed in triplicate. Germination rate (%) was calculated as follows:

$$\mathrm{Germination}\ \mathrm{rate}=\frac{\mathrm{No}.\mathrm{of}\ \mathrm{seeds}\ \mathrm{germinated}}{\mathrm{Total}\ \mathrm{no}.\mathrm{of}\ \mathrm{seeds}\ \mathrm{tested}}\times 100\%$$

Simple vigor index was calculated as follows:

$$Simple\;vigor\;index\:=\:Germination\;rate\;(\%)\:\times\:Average\;bud\;length\;(cm)$$

The average germination rate of three replicates was calculated. Statistical analysis was carried out using Student’s *t*-test.

### Aging treatment

Healthy rice seeds of the same size and at the same maturity level were exposed to high temperature (43 ± 2 °C) and relative humidity (RH; 100%) for 8 days, and then tested for germination as described above.

To determine the anti-aging ability of *Arabidopsis* seeds, dry mature seeds stored at 4 °C for more than 2 days were first exposed to high temperature (42 °C) and RH (100%) for 6 days and then surface-sterilized in 70% ethanol for 2 min, followed by soaking in 10% bleach for 20 min, and then rinsed extensively in sterile water for at least five times. The germination of aged and unaged (control) seeds was tested as described by Chen et al. [44].

### RNA extraction, Degradome library construction, sequencing and data analysis

Total RNA was extracted from the embryos of unaged and artificially aged WT, *OE164c* and *MIM164c* seeds as described previously [10]. The RNA integrity of each sample was evaluated using an Agilent Bioanalyzer 2100 (Agilent), and samples with A_260_/A_280_ values of 1.8–2.1 were used for degradome sequencing. Six degradome libraries were constructed. Briefly, 20 μg of total RNA of each sample was subjected to two rounds of purification using poly-T oligo-attached magnetic beads to purify poly(A) RNA. Then, RNA ligase was used to ligate adapters to the 5′ end of the 3′ cleavage product of the mRNA. Reverse transcription was performed using a 3′-adapter random primer to synthesize the first strand of cDNA, which was size-selected using AMPureXP beads. Then, cDNA was PCR amplified under the following conditions: initial denaturation at 95 °C for 3 min, followed by 15 cycles of denaturation at 98 °C for 15 s, annealing at 60 °C for 15 s and extension at 72 °C for 30 s, and a final extension at 72 °C for 5 min. The average insert size of the final cDNA library was 200–400 bp. Finally, 50-bp single-end sequencing was performed on the Illumina Hiseq2500 platform, according to the manufacturer’ s instructions (LC Bio, Hangzhou, China). The quality of the sequencing data was presented in Sanger format, which encodes quality scores ranging from 0 to 93 in ASCII characters 33 to 126; the higher the quality score, the smaller the error rate. A publicly available software package, CleaveLand 3.0, was used for analyzing the sequencing data generated. The comparable pair sequence obtained was compared with the cDNA sequence of the rice cultivar ‘Nipponbare’ to generate a degradation density file. The corresponding target mRNAs that pair with small RNA sequences of the rice cultivar ‘Nipponbare’ were predicted using TargetFinder. The reads of each library were normalized by TPB (Transcript per billion counts), and normalized expression = (actual mRNA count/total count of clean reads) × 1,000,000,000. The TPB value indicates the abundance of the transcript being cleaved. To facilitate comparisons of the abundance of target transcripts being cleaved and functional cluster analysis of the transcripts in unaged and artificially aged WT, *MIM164c*, and *OE164c* seeds, zero mean normalization was carried out for the TPB value of the degradome transcripts common to the six libraries (Z-score = $\frac{\left(\mathrm{x}-\upmu \right)}{\upsigma}$, x represents the TPB value, μ represents the average value of the TPB in six libraries, and σ represents the standard deviation). The gene ontology (GO) functional annotation and the Kyoto Encyclopedia of Genes and Genomes (KEGG) pathways of the degradome transcripts were performed based on the GO database (ftp://ftp.ncbi.nih.gov/gene/DATA/gene2go.gz, last modified: 2016–04) and KEGG database (http://www.genome.jp/kegg, release: 2016–05). GO functional classifications were performed using the method described in Huang et al. [11].

The Uniport website (Last modified: 2019–02) was used to predict the subcellular localization of the target transcripts.

### Gene interaction prediction

A gene–gene interaction network was constructed using the following genes: target genes corresponding to degradome transcripts; genes corresponding to the six functional categories of rice seed vigor-related DEPs [11]; and genes corresponding to the DEPs among artificially aged WT, *MIM164c* and *OE164c* seeds (fold-change [FC] > 1.3 or < 1/1.3), which were identified by proteome analysis using the method described in Huang et al. [11]. These genes were inputted into STRING (Version: 11.0), a gene interaction prediction database, to predict the interaction network among the target genes regulated by miRNAs and other functional genes.

### Real-time quantitative reverse transcription PCR (RT-qPCR)

Total RNA was extracted from the embryos of unaged and artificially aged WT, *MIM164c* and *OE164c* seeds using the TransGen TransZol Plant kit (Vazyme, Nanjing, China). Stem-loop reverse transcription of miRNAs was performed using the Vazyme miRNA 1st Strand cDNA Synthesis Kit (Vazyme). RT-qPCR was performed using the miRNA Universal SYBR qPCR Master Mix kit (Vazyme), with *U6* as the internal reference.

To detect the expression of miRNA target genes in seeds, the same total RNA samples (as used above) were reversely transcribed using the Vazyme HiScript II Q RT SuperMix for qPCR (+gDNA wiper) kit (Vazyme). Then, RT-qPCR analysis was performed using the ChamQ Universal SYBR qPCR Master Mix kit (Vazyme), with *Osactin* as the internal reference.

Primers used for RT-qPCR are listed in Table S1.

### Subcellular localization analysis

To determine the subcellular localization of the miR5075 target protein Os02g0817500, the cDNA of *Os02g0817500* (without the stop codon) was cloned into the pCUbi1390 vector. Primers are listed in Table S1. The resultant construct or empty pCUbi1390 vector (positive control) was transformed into rice protoplasts via polyethylene glycol (PEG)-mediated transformation [65]. Green fluorescence protein signals were visualized using a fluorescence microscope (German Zeiss LSM880).

### Yeast two-hybrid (Y2H) assay

Two Y2H libraries (one for the nucleus system, and the other for the membrane system) to identify novel protein interactions were constructed by Oebiotech (Shanghai, China) using total RNA extracted from the embryos of ‘Kasalath’ and ‘Nipponbare’ seeds subjected to artificial aging for 0, 8, and 14 days and to germination conditions for 1 day, respectively. To perform the Y2H assay, the *Os02g0817500* cDNA was cloned into the pGBKT7 and pBT3-N vectors. Primers are listed in Table S1. The recombinant constructs were separately transformed into the yeast strains Y2H and NMY51. Both libraries were screened using Os02g0817500 protein as the bait, according to the manufacturer’s instructions (Invitrogen).

## Supplementary Information

**Additional file 1: Table S1.** List of primers used in this study. **Table S2.** List of miRNAs and target transcripts identified by degradome sequencing, along with the TPB value of the degradome transcript in the six libraries. **Table S3.** Statistics of the number of target genes in the degradome dataset or transcript database annotated by the GO terms and KEGG pathways. **Table S4.** Subcellular localization of different types of degradome transcripts. **Table S5.** A STRING-based network showing the interactions among nodes (i.e., genes or proteins).

**Additional file 2: Figure S1.** Category “Biological process” of Gene Ontology (GO) enrichments of target genes corresponding to the unique to unaged and artificially aged WT, *MIM164c*, and *OE164c* seeds. The darker the color, the more the number of target genes enriched. A1, A2, and A3 indicate unaged WT, *MIM164c* and *OE164c* seeds, respectively; B1, B2, and B3 represent artificially aged WT, *MIM164c*, and *OE164c* seeds, respectively. **Figure S2.** Category “Molecular function” of Gene Ontology (GO) enrichments of target genes corresponding to the unique to unaged and artificially aged WT, *MIM164c*, and *OE164c* seeds. The darker the color, the more the number of target genes enriched. A1, A2, and A3 indicate unaged WT, *MIM164c* and *OE164c* seeds, respectively; B1, B2, and B3 represent artificially aged WT, *MIM164c*, and *OE164c* seeds, respectively. **Figure S3.** Category “Cellular component” of Gene Ontology (GO) enrichments of target genes corresponding to the unique to unaged and artificially aged WT, *MIM164c*, and *OE164c* seeds. The darker the color, the more the number of target genes enriched. A1, A2, and A3 indicate unaged WT, *MIM164c* and *OE164c* seeds, respectively; B1, B2, and B3 represent artificially aged WT, *MIM164c*, and *OE164c* seeds, respectively.

**Additional file 3: Figure S4.** The prediction of subcellular distributions and proportions of proteins encoded by miRNA target genes corresponding to the degradome transcripts. The value in the outer circle represents the percentage of the number of degradome transcripts of each category, the value in the inner circle represents the number of degradome transcripts of each category.

**Additional file 4: Figure S5.** Source data for Fig. 9A.

**Additional file 5: Figure S6.** Red frame displayed the source data for Fig. 10A.

**Additional file 6: Figure S7.** RT-qPCR analysis of the expression levels of plant hormone pathway related genes. Data represent mean ± standard deviation (SD; *n* = 3). Significant differences among samples were determined using Student’s *t*-test (*P < 0.05, **P < 0.01, ***P < 0.001).

## Data Availability

All data supporting the findings were contained in the manuscript and its supplementary files except the raw DGD-seq data and the mass spectrometry proteomics data analysed during the current study. The raw DGD-seq data have been deposited in Sequence Read Archive (SRA) (http://www.ncbi.nlm.nih.gov/sra/PRJNA801424), the accession number is PRJNA801424. The mass spectrometry proteomics data have been deposited in the ProteomeXchange Consortium via the PRIDE partner repository with the dataset identifier PXD031352 (http://proteomecentral.proteomexchange.org/cgi/GetDataset?ID=PXD031352) (Reviewer account details: Username: reviewer_pxd031352@ebi.ac.uk; Password: IEbtedf7). The datasets and materials used and/or analysed during the current study are available from the corresponding author on reasonable request.

## Abbreviations

ABA: Abscisic acid
AKR: Aldo/keto reductase
ARF: Auxin response factor
BR: Brassinosteroid
DEP: Differentially expressed protein
DGD-seq: Degradome sequencing
ER: Endoplasmic reticulum
ERAD: Endoplasmic reticulum-associated degradation
EREBP: Ethylene-responsive element binding protein
FC: Fold-change
GO: Gene Ontology
IAA: Indoleacetic acid
KEGG: Kyoto Encyclopedia of Genes and Genomes
*MIM164c*: Mimick *miR164c* (miR164c-silenced) line
miRNA: MicroRNAs
MS: Murashige and Skoog
*OE-*1/*OE-*2: *Os02g0817500* overexpression lines
*OE164c*: MiR164c-overexpression line
PCD: Programmed cell death
PEG: Polyethylene glycol
PR: Pathogenesis-related
QTL: Quantitative trait locus
RH: Relative humidity
ROS: Reactive oxygen species
RT-PCR: Reverse transcription polymerase chain reaction
RT-qPCR: Real-time quantitative polymerase chain reaction
SA: Salicylic acid
SL: Strigolactone
TCA: Tricarboxylic acid
TOC: Total organic carbon
TPB: Transcripts per billion counts
WT: Wild-type
Y2H: Yeast two-hybrid
