# Correction: Degradome sequencing reveals an integrative miRNA-mediated gene interaction network regulating rice seed vigor

**DOI:** 10.1186/s12870-022-03700-y

**Published:** 2022-06-28

**Authors:** Shiqi Zhou, Kerui Huang, Yan Zhou, Yingqian Hu, Yuchao Xiao, Ting Chen, Mengqi Yin, Yan Liu, Mengliang Xu, Xiaocheng Jiang

**Affiliations:** 1 College of Life Sciences, Hunan Normal University, Changsha, 410081 China; 2 Hunan Province Key Laboratory of Crop Sterile Germplasm Resource Innovation and Application, Changsha, 410081 China; 3 College of Life and Environmental Sciences, Hunan University of Arts and Science, Changde, 415000 China

**Correction: BMC Plant Biol 22, 269 (2022)**

**https://doi.org/10.1186/s12870-022-03645-2**

Following publication of the original article [1], authors identified an error found in the affiliations. There is an omission in the affiliation of one of the co-authors, Kerui Huang, who si affiliated to:

1College of Life Sciences, Hunan Normal University, Changsha, 410081, China

2Hunan Province Key Laboratory of Crop Sterile Germplasm Resource Innovation and Application, Changsha, 410081, China

3College of Life and Environmental Sciences, Hunan University of Arts and Science, Changde, 415000, China

Mengliang Xu and Xiaocheng Jiang are affiliated to:

1College of Life Sciences, Hunan Normal University, Changsha, 410081, China

2Hunan Province Key Laboratory of Crop Sterile Germplasm Resource Innovation and Application, Changsha, 410081, China

The correction does not have any effect on the results or conclusions of the paper. The original article has been corrected.
