# Supplementary figures and images for: Degradome sequencing reveals an integrative miRNA-mediated gene interaction network regulating rice seed vigor
Source: BMC Plant Biol. 2022 Jun 1;22:269. doi: 10.1186/s12870-022-03645-2 (PMC9158300; doi:10.1186/s12870-022-03645-2)

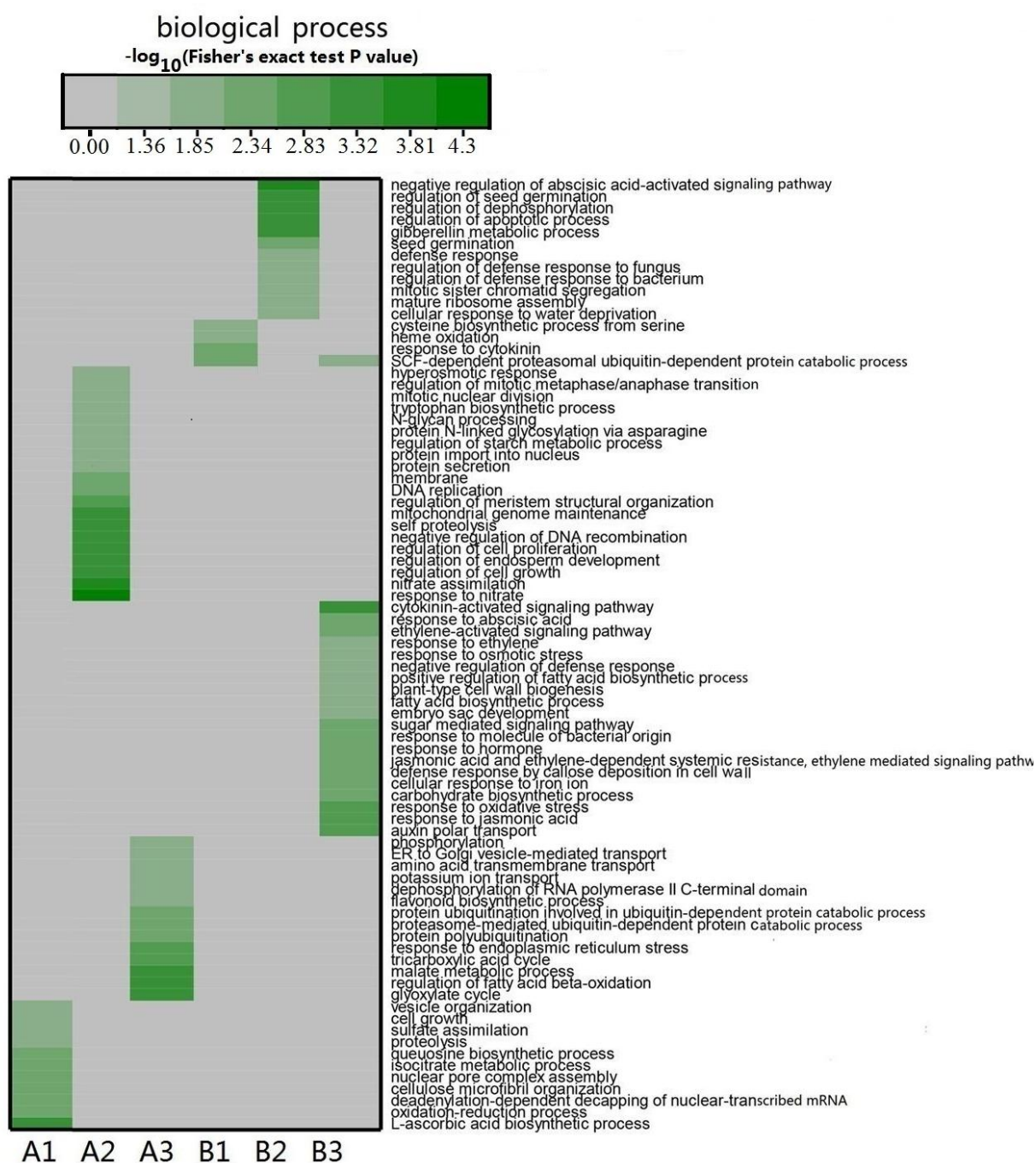

Supplement: Supplementary file 2 — Additional file 2: Figure S1. Category “Biological process” of Gene Ontology (GO) enrichments of target genes corresponding to the unique to unaged and artificially aged WT, MIM164c, and OE164c seeds. The darker the color, the more the number of target genes enriched. A1, A2, and A3 indicate unaged WT, MIM164c and OE164c seeds, respectively; B1, B2, and B3 represent artificially aged WT, MIM164c, and OE164c seeds, respectively. Figure S2. Category “Molecular function” of Gene Ontology (GO) enrichments of target genes corresponding to the unique to unaged and artificially aged WT, MIM164c, and OE164c seeds. The darker the color, the more the number of target genes enriched. A1, A2, and A3 indicate unaged WT, MIM164c and OE164c seeds, respectively; B1, B2, and B3 represent artificially aged WT, MIM164c, and OE164c seeds, respectively. Figure S3. Category “Cellular component” of Gene Ontology (GO) enrichments of target genes corresponding to the unique to unaged and artificially aged WT, MIM164c, and OE164c seeds. The darker the color, the more the number of target genes enriched. A1, A2, and A3 indicate unaged WT, MIM164c and OE164c seeds, respectively; B1, B2, and B3 represent artificially aged WT, MIM164c, and OE164c seeds, respectively. [file 12870_2022_3645_MOESM2_ESM.zip › Figure S1.pdf]

molecular function  
 $-\log_{10}(\text{Fisher's exact test P value})$

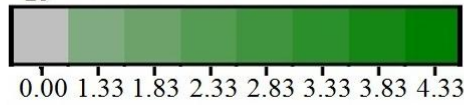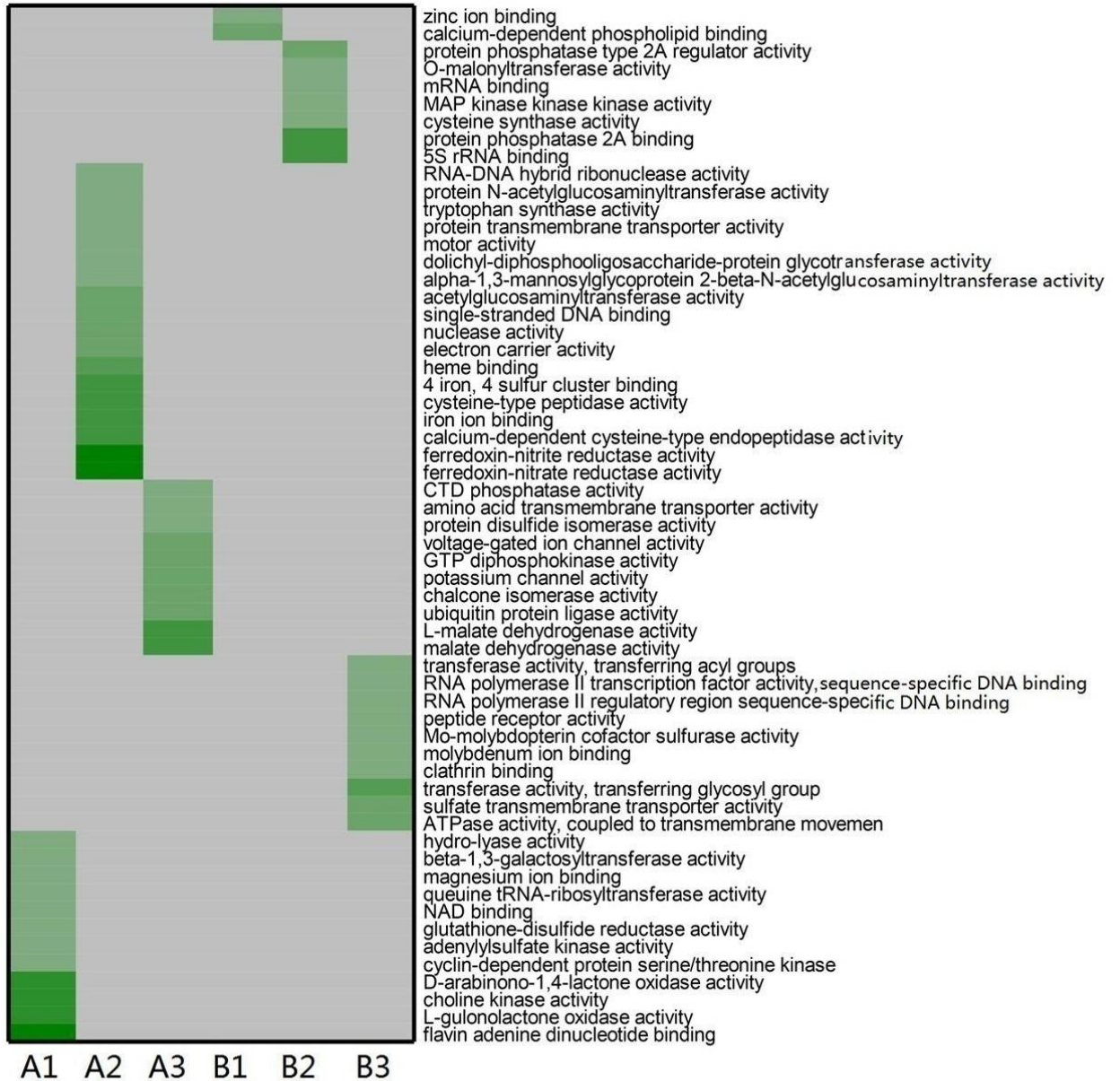

Supplement: Supplementary file 2 — Additional file 2: Figure S1. Category “Biological process” of Gene Ontology (GO) enrichments of target genes corresponding to the unique to unaged and artificially aged WT, MIM164c, and OE164c seeds. The darker the color, the more the number of target genes enriched. A1, A2, and A3 indicate unaged WT, MIM164c and OE164c seeds, respectively; B1, B2, and B3 represent artificially aged WT, MIM164c, and OE164c seeds, respectively. Figure S2. Category “Molecular function” of Gene Ontology (GO) enrichments of target genes corresponding to the unique to unaged and artificially aged WT, MIM164c, and OE164c seeds. The darker the color, the more the number of target genes enriched. A1, A2, and A3 indicate unaged WT, MIM164c and OE164c seeds, respectively; B1, B2, and B3 represent artificially aged WT, MIM164c, and OE164c seeds, respectively. Figure S3. Category “Cellular component” of Gene Ontology (GO) enrichments of target genes corresponding to the unique to unaged and artificially aged WT, MIM164c, and OE164c seeds. The darker the color, the more the number of target genes enriched. A1, A2, and A3 indicate unaged WT, MIM164c and OE164c seeds, respectively; B1, B2, and B3 represent artificially aged WT, MIM164c, and OE164c seeds, respectively. [file 12870_2022_3645_MOESM2_ESM.zip › Figure S2.pdf]

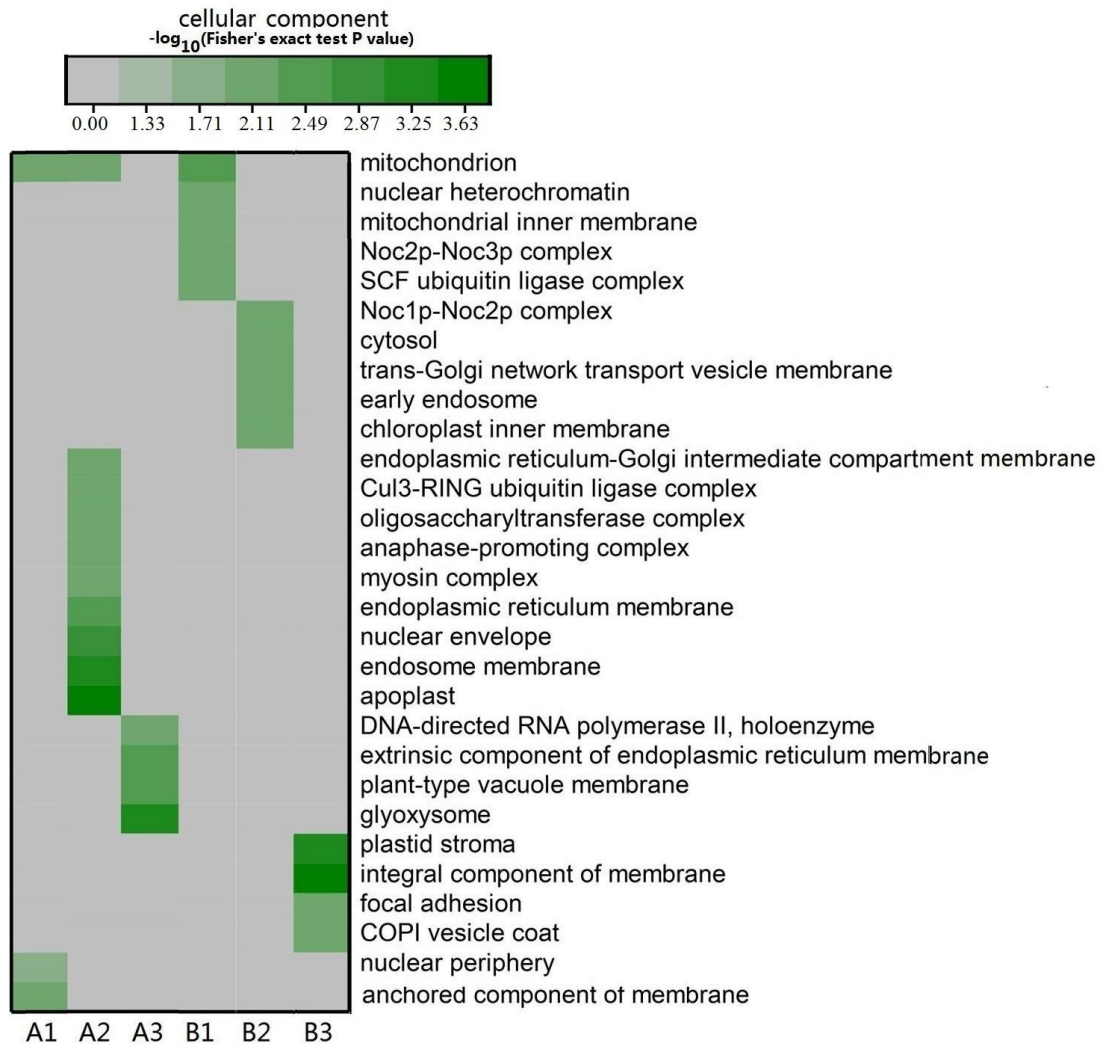

Supplement: Supplementary file 2 — Additional file 2: Figure S1. Category “Biological process” of Gene Ontology (GO) enrichments of target genes corresponding to the unique to unaged and artificially aged WT, MIM164c, and OE164c seeds. The darker the color, the more the number of target genes enriched. A1, A2, and A3 indicate unaged WT, MIM164c and OE164c seeds, respectively; B1, B2, and B3 represent artificially aged WT, MIM164c, and OE164c seeds, respectively. Figure S2. Category “Molecular function” of Gene Ontology (GO) enrichments of target genes corresponding to the unique to unaged and artificially aged WT, MIM164c, and OE164c seeds. The darker the color, the more the number of target genes enriched. A1, A2, and A3 indicate unaged WT, MIM164c and OE164c seeds, respectively; B1, B2, and B3 represent artificially aged WT, MIM164c, and OE164c seeds, respectively. Figure S3. Category “Cellular component” of Gene Ontology (GO) enrichments of target genes corresponding to the unique to unaged and artificially aged WT, MIM164c, and OE164c seeds. The darker the color, the more the number of target genes enriched. A1, A2, and A3 indicate unaged WT, MIM164c and OE164c seeds, respectively; B1, B2, and B3 represent artificially aged WT, MIM164c, and OE164c seeds, respectively. [file 12870_2022_3645_MOESM2_ESM.zip › Figure S3.pdf]

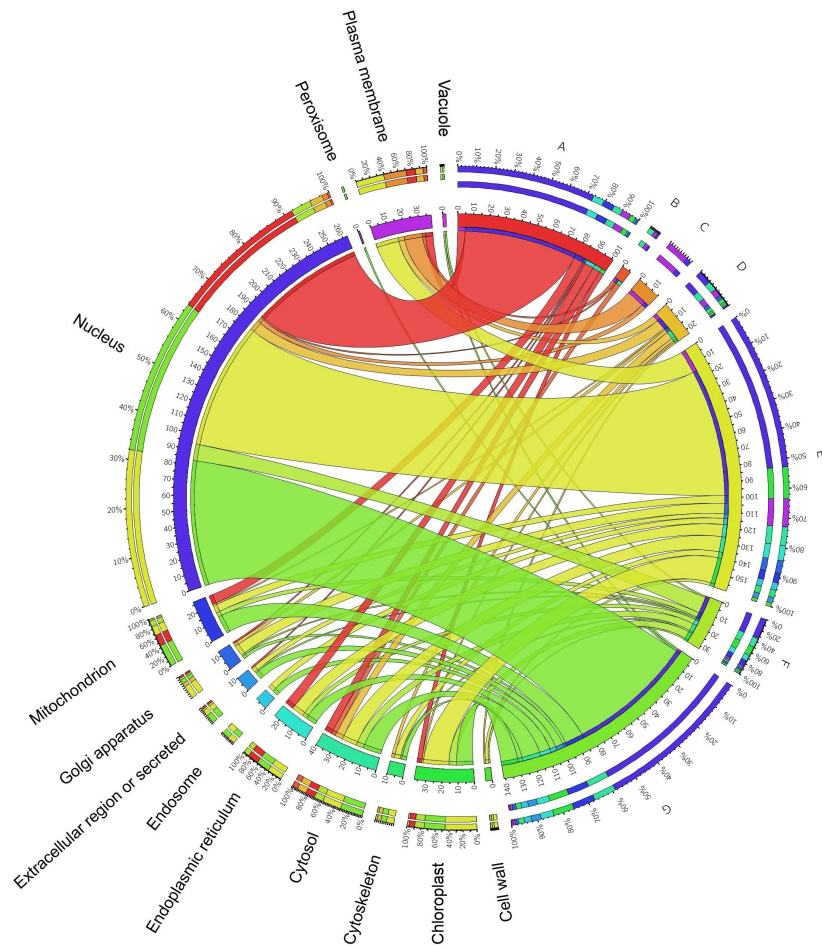

Supplement: Supplementary file 3 — Additional file 3: Figure S4. The prediction of subcellular distributions and proportions of proteins encoded by miRNA target genes corresponding to the degradome transcripts. The value in the outer circle represents the percentage of the number of degradome transcripts of each category, the value in the inner circle represents the number of degradome transcripts of each category. [file 12870_2022_3645_MOESM3_ESM.pdf]

WT

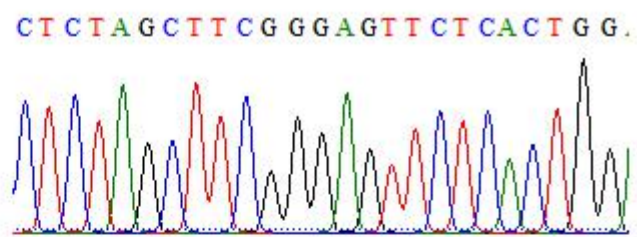

*Os02g0817500-1*

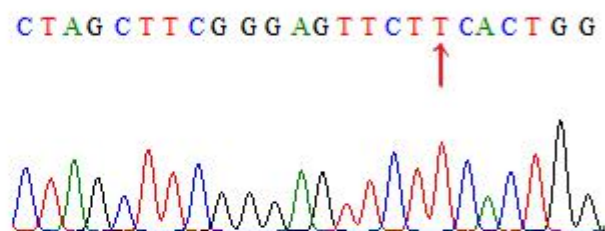

*Os02g0817500-2*

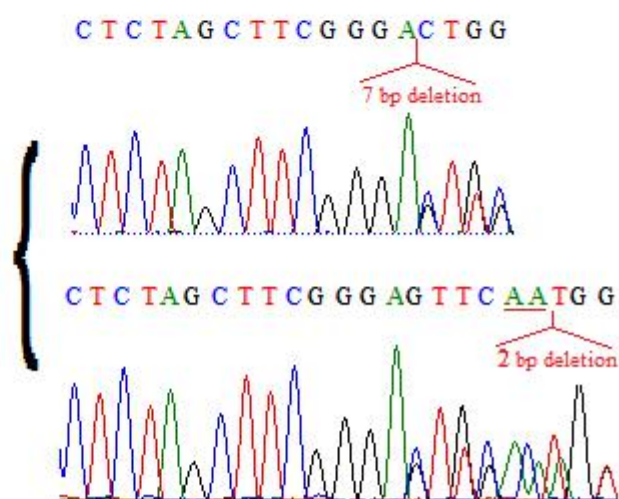

Supplement: Supplementary file 4 — Additional file 4: Figure S5. Source data for Fig. 9A. [file 12870_2022_3645_MOESM4_ESM.pdf]

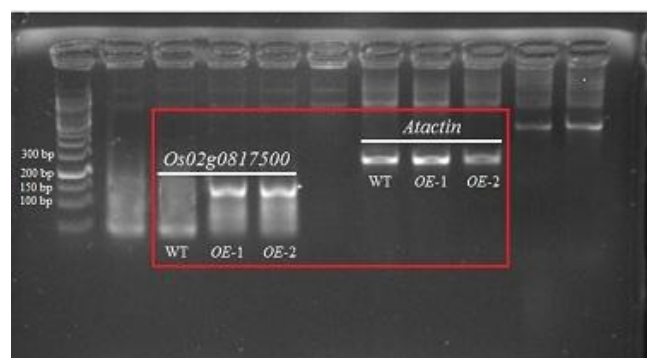

Supplement: Supplementary file 5 — Additional file 5: Figure S6. Red frame displayed the source data for Fig. 10A. [file 12870_2022_3645_MOESM5_ESM.pdf]

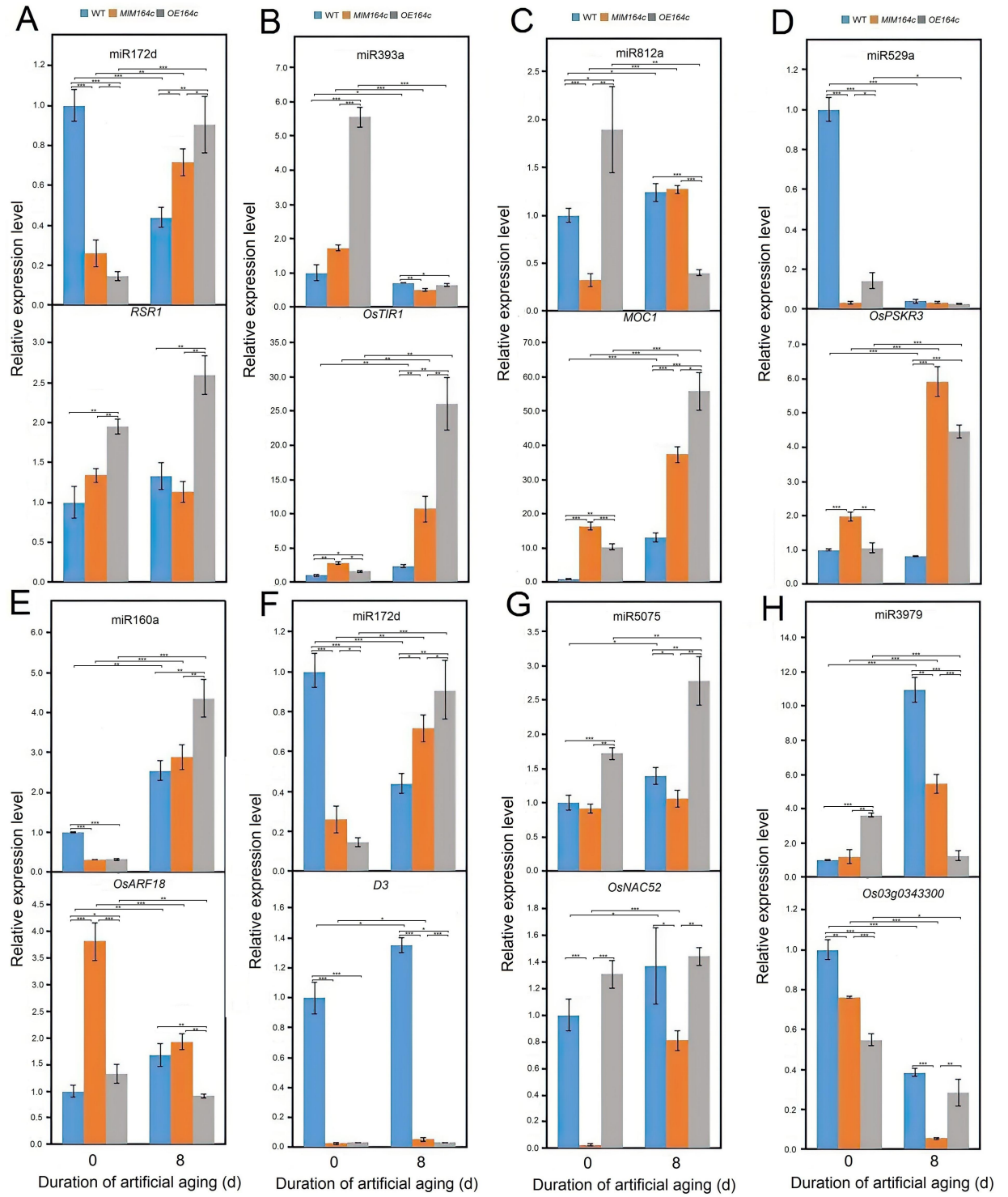

Supplement: Supplementary file 6 — Additional file 6: Figure S7. RT-qPCR analysis of the expression levels of plant hormone pathway related genes. Data represent mean ± standard deviation (SD; n = 3). Significant differences among samples were determined using Student’s t-test (*P < 0.05, **P < 0.01, ***P < 0.001). [file 12870_2022_3645_MOESM6_ESM.pdf]
